# Characteristics and impact of infiltration of B-cells from systemic sclerosis patients in a 3D healthy skin model

**DOI:** 10.3389/fimmu.2024.1373464

**Published:** 2024-08-09

**Authors:** Mathilde Le Maître, Thomas Guerrier, Aurore Collet, Mehdi Derhourhi, Jean-Pascal Meneboo, Bénédicte Toussaint, Amélie Bonnefond, Céline Villenet, Shéhérazade Sebda, Antonino Bongiovanni, Meryem Tardivel, Myriam Simon, Manel Jendoubi, Blanche Daunou, Alexis Largy, Martin Figeac, Sylvain Dubucquoi, David Launay

**Affiliations:** ^1^ Univ. Lille, Inserm, CHU Lille, U1286 - INFINITE - Institute for Translational Research in Inflammation, Lille, France; ^2^ CHU Lille, Institut d’Immunologie, Pôle de Biologie Pathologie Génétique, Lille, France; ^3^ Inserm UMR1283, CNRS UMR8199, European Genomic Institute for Diabetes (EGID), Institut Pasteur de Lille, Lille University Hospital, Lille, France; ^4^ Université de Lille, Lille, France; ^5^ Univ. Lille, CNRS, Inserm, CHU Lille, Institut Pasteur de Lille, US 41 - UAR 2014 - PLBS, Lille, France; ^6^ Department of Metabolism, Digestion and Reproduction, Imperial College London, London, United Kingdom; ^7^ Service de Médecine Interne et d’Immunologie Clinique, Centre de Référence Des Maladies Auto-Immunes et Systémiques Rares du Nord et Nord-Ouest de France (CeRAINO), CHU Lille, Lille, France

**Keywords:** B-cell, systemic sclerosis, skin, 3D coculture, fibrosis, fibroblast

## Abstract

**Introduction:**

In systemic sclerosis (SSc), B-cells are activated and present in the skin and lung of patients where they can interact with fibroblasts. The precise impact and mechanisms of the interaction of B-cells and fibroblasts at the tissular level are poorly studied.

**Objective:**

We investigated the impact and mechanisms of B-cell/fibroblast interactions in cocultures between B-cells from patients with SSc and 3-dimensional reconstituted healthy skin model including fibroblasts, keratinocytes and extracellular matrix.

**Methods:**

The quantification and description of the B-cell infiltration in 3D cocultures were performed using cells imagery strategy and cytometry. The effect of coculture on the transcriptome of B-cells and fibroblasts was studied with bulk and single-cell RNA sequencing approaches. The mechanisms of this interaction were studied by blocking key cytokines like IL-6 and TNF.

**Results:**

We showed a significant infiltration of B-cells in the 3D healthy skin model. The amount but not the depth of infiltration was higher with B-cells from SSc patients and with activated B-cells. B-cell infiltrates were mainly composed of naïve and memory cells, whose frequencies differed depending on B-cells origin and activation state: infiltrated B-cells from patients with SSc showed an activated profile and an overexpression of immunoglobulin genes compared to circulating B-cells before infiltration. Our study has shown for the first time that activated B-cells modified the transcriptomic profile of both healthy and SSc fibroblasts, toward a pro-inflammatory (TNF and IL-17 signaling) and interferon profile, with a key role of the TNF pathway.

**Conclusion:**

B-cells and 3D skin cocultures allowed the modelization of B-cells infiltration in tissues observed in SSc, uncovering an influence of the underlying disease and the activation state of B-cells. We showed a pro-inflammatory effect on skin fibroblasts and pro-activation effect on infiltrating B-cells during coculture. This reinforces the role of B-cells in SSc and provide potential targets for future therapeutic approach in this disease.

## 1 Introduction

Systemic sclerosis (SSc) is the most severe systemic autoimmune disease characterized by a widespread fibrosis affecting the skin and internal organs. The pathophysiology of SSc is characterized by a combination of vasculopathy, dysregulated activation of both innate and adaptive immunities, and uncontrolled fibrogenesis. Treatment options for SSc primarily encompass vasoactive agents, immunosuppressants, and antifibrotic therapies. However, the disease remains incurable, with only limited efficacy observed in current treatment strategy, underscoring the urgent necessity to expand the therapeutic armamentarium.

Among the different immune cells implicated in the pathophysiology of SSc, the presence of autoantibodies and hypergammaglobulinemia has long suggested a role of B-cells. More recently, the role of B-cells has been reinforced by data on their altered homeostasis and function in SSc, the development of animal models of immunization (1) and the rapid expansion of anti-B-cell biotherapies suggesting their efficacy in the disease (2). In a recent review, we further showed that B-cell are found in the skin of patients with SSc and in animal models (2–7). Interestingly, in skin biopsies analyzed using DNA microarray screening for more than 12,000 genes (3, 8, 9), among almost 3000 genes differentially expressed between SSc patients and healthy controls, the authors identified a cluster of B-cell-associated genes (mostly immunoglobulin (Ig) genes) highly upregulated in a subset of patients with a diffuse cutaneous SSc. Yet, the exact pathogenic role of B-cells in SSc has still to be fully determined. B-cells can produce proinflammatory and profibrotic cytokines as well as autoantibodies, which could impact key target cells like fibroblasts or endothelial cells (2). Rituximab depletion of B-cells in the skin and peripheral blood is associated with a decreased production of reactive oxygen species and fibrosis markers (collagen, α-SMA) by skin fibroblasts (4). However, the direct cell-cell interaction of B-cells with these targeted cells has only been scarcely studied. François et al. in 2-dimensional cocultures, showed that B-cells were able to increase the collagen production of SSc fibroblasts (10). Indeed, when SSc fibroblasts were cocultured with healthy control B-cells stimulated by BAFF (inducing the secretion by B-cells of pro-fibrotic cytokines such as IL-6, CCL-2, TGF-β1), they produced tissue inhibitor of metalloprotease-1 (TIMP-1), collagens, and α-smooth muscle actin (α-SMA) (10). Moreover, this phenomenon was weaker when the cells were cocultured in Transwell^©^ inserts, indicating a participation of direct intercellular contacts in addition to soluble mediators.

There are many unanswered questions concerning the B-cell/fibroblast interactions including the impact on both fibroblasts as well as on B-cells and the mechanisms of these interactions. Moreover, the 2D cultures have some limits as they do not include other important actors like keratinocytes (11).

To fill these gaps, we designed the present study to investigate the impact and mechanisms of B-cell/fibroblast interactions on both cell types, in cocultures in a healthy 3-dimensional reconstituted skin model including fibroblasts, keratinocytes and extracellular matrix. Using a multimodal strategy, including cell imagery, flow cytometry, and bulk and single-cell transcriptomic approaches, we showed that B-cells can infiltrate the dermal layer of the reconstituted skin, especially when B-cells are previously primed *in vitro*. Blood B-cells collected from SSc patients can also significantly infiltrate skin even without previous cells priming. Infiltrated B-cells were markedly activated and overexpressing immunoglobulin genes.

## 2 Material and methods

### 2.1 Human samples

Healthy skin samples were obtained from surgery waste at the department of aesthetic surgery of Lille hospital. Skin biopsies and blood samples were obtained from patients with SSc from the department of internal medicine of Lille hospital. Blood samples from healthy donors were obtained from the Etablissement Français du Sang (Lille, France). Patient data is available in **Table 1**.

All human samples were acquired in compliance with institutional regulations. The protocol was approved by the Regional Ethics Committee for Patient Protection (CPP N° ID-RCB 2021-A03137–34), and all participants gave their written, informed consent. Patient data is available in **Table 1**. SSc patients were included when they fulfilled the following criteria: 1. They fulfilled the ACR/EULAR 2013 Criteria for SSc 2. They had no immunosuppressive treatment within the year before sampling, but 10 mg or less of prednisone or equivalent was accepted.

**Table 1:** Clinical characteristics of patients with systemic sclerosis.

Patient	Age (years)	Sex	Type of SSc	Duration of disease (years)	Type of autoantibodies	Presence of ILD	Corticosteroid treatment (per day)
1	73	H	diffuse	5	anti-centromere	yes	no
2	55	F	diffuse	9	anti-RNApol III	no	no
3	52	F	diffuse	12	anti-centromere	no	no
4	62	F	diffuse	4	anti-centromere	yes	no
5	47	F	diffuse	5	anti-RNApol III	no	5 mg
6	57	F	diffuse	20	anti-topoisomerase I	yes	10 mg
7	46	F	diffuse	34	anti-topoisomerase I	yes	no
8	54	F	limited	10	anti-topoisomerase I	yes	10 mg
9	33	H	limited	1	anti-topoisomerase I	no	10 mg
10	72	F	limited	4	anti-topoisomerase I	yes	no
11	62	F	diffuse	24	anti-centromere	no	no
12	59	H	diffuse	6	anti-topoisomerase I	no	5 mg
13*	58	F	diffuse	27	anti-topoisomerase I	yes	no
14	63	H	diffuse	5	none	no	no
15	47	F	limited	13	anti-topoisomerase I	yes	no
16	60	F	diffuse	4	anti-topoisomerase I, anti-RNApol III	yes	4 mg
17	54	F	diffuse	37	anti-RNApol III	yes	5 mg
18	38	H	diffuse	26	anti-RNApol III	yes	No
19	42	F	diffuse	9	anti-RNApol III	no	7 mg
20	50	M	diffuse	2	anti-topoisomerase I	yes	No
21*	28	F	diffuse	2	anti-topoisomerase I	yes	No
22	77	F	limited	14	anti-topoisomerase I	no	No
23	28	F	diffuse	4	anti-RNApol III	no	No
24	66	M	diffuse	0.5	anti-topoisomerase I	yes	9 mg
25*	36	F	diffuse	9	anti-topoisomerase I	yes	5 mg
26	47	F	diffuse	18	anti-topoisomerase I	yes	No
27	36	F	limited	3	anti-centromere	no	5 mg
28	43	F	limited	16	anti-PM-Scl	no	5 mg
29	74	F	diffuse	1	ANA without specificity	no	10 mg
30	49	F	diffuse	31	ANA without specificity	yes	no
31	46	F	diffuse	1	anti-topoisomerase I	no	no

Patients whose B-cells were used for single-cell analysis are labeled with an asterisk. Duration of the disease was calculated from apparition of the first signs of SSc besides Raynaud syndrome. ILD, Interstitial Lung Disease. ANA, antinuclear antibodies; RNApol III, RNA polymerase III.

### 2.2 B-cells purification

PBMCs were extracted from blood using Ficoll density gradient, and used after cryopreservation. Frozen PBMC were thawed, and B-cells were purified using the EasySep™ Negative Human B-cell Isolation Kit (STEMCELL Technologies, Vancouver, Canada) per the manufacturer’s instructions.

### 2.3 Cell culture and 2D cocultures

Dermal primary fibroblasts were extracted from healthy and SSc skin samples with explant culture method following EUSTAR guidelines for biobanking (12) or enzymatic dissociation using the Whole Skin Dissociation Kit, human (Miltenyi, Bergisch Gladbach, Germany). Cells were frozen at several passages preserved in DMEM 20% FCS 10% DMSO at -150°C.

For 2D cocultures, 40 000 healthy or SSc fibroblasts between P4 and P6 were seeded in a 24-well plate and incubated overnight at 37°C and 5% CO_2_ in RPMI deprived of FCS. The day after, medium was replaced with AIM (Gibco/ThermoFisher Scientific, Waltham, MA, USA) with or without the following cytokine inhibitors or isotype antibodies at 10 µg/ml: Tocilizumab (RoActemra^®^; Roche Pharma, Grenzach-Wyhlen, Germany), Infliximab (Remicade^®^; MSD, Puteaux, France), Human Lymphotoxin-α/TNF-β antibodies (AF-211-NA; R&D Systems, Minneapolis, MN, USA), TD139 (TargetMol, Wellesley Hills, MA, USA), Normal Goat IgG Control antibodies (AB-108-C; R&D Systems, Minneapolis, MN, USA), Ultra-LEAF™ Purified Human IgG1 Isotype Control Recombinant antibodies (Biolegend, San Diego, CA, USA). B lymphocytes from healthy donors or SSc patients were placed either on top of fibroblasts or separated from them by a 0.4 µm transwell. A mix of stimulation molecules, including AffiniPure F(ab’)2 Fragment Goat Anti-Human IgA + IgG + IgM (10 µg/ml; Jackson Immunology, West Grove, PA, USA), recombinant human CD40 Ligand/TNFSF5 (Histidine-tagged) (50 ng/ml; R&D Systems, Minneapolis, MN, USA), and His Tag monoclonal antibodies (Clone AD1.1.10) (5 µg/ml; R&D Systems, Minneapolis, MN, USA), was added on top of B-cells when necessary. Cocultures were then incubated during 48h at 37°C and 5% CO_2_.

After incubation, supernatants were collected, and cells were trypsinized during 5 min and removed from wells. For wells without inserts, cell mixes were sorted by FACS to collect viable fibroblasts.

### 2.4 3D cocultures

3-dimensional healthy full thickness skin models T-skin™ were purchased at day 11 from Episkin (Lyon, France) and maintained in culture on inserts in 6-well plates with GDA3F+ medium (Episkin, Lyon, France) changed every other day.

For activated B-cells, during 24 to 48h prior to coculture, B-cells were incubated at 1M per ml with a mix of AffiniPure F(ab’)2 Fragment Goat Anti-Human IgA + IgG + IgM (10 µg/ml; Jackson Immunology, West Grove, PA, USA), Recombinant Human CD40 Ligand/TNFSF5 (Histidine-tagged) (50 ng/ml; R&D Systems, Minneapolis, MN, USA), His Tag monoclonal antibodies (Clone AD1.1.10) (5 µg/ml; R&D Systems, Minneapolis, MN, USA) and CpG ODN 2006 (10 µg/ml; InvivoGen, San Diego, CA, USA).

A maximum of 500 000 B-cells were placed at the center of a 6-well 0.1 µm insert in 50µl or less of RPMI FCS 10%. The skin model was then gently placed on top of the B-cell deposit. Cocultures were incubated during 4 to 5 days in GDA3F+ medium at 37°C and 5% CO_2_.

### 2.5 Confocal microscopy

For confocal microscopy, B-cells and 3D skin models removed from their insert were incubated prior to coculture with CellTracker™ Red CMTPX (ThermoFisher Scientific, Waltham, MA, USA) at 10 µM during 30 min in RPMI SVF 10% and CellTracker ™DeepRed (ThermoFisher Scientific, Waltham, MA, USA) at 8 µM during 45 min in GDA3F+ medium respectively, at 37°C and 5% CO_2_ and protected from light. B-cells were then rinsed with medium and centrifuged two times at 400G for 5 min, while skin models were rinsed in 5ml of PBS 1X during 15 min under agitation.

After coculture with B-cells, skin models were fixed in 4% paraformaldehyde for 24h, then underwent successive 3h baths of increasing concentrations (30%, 50%, 70%, 97%) of 2,2’-Thiodiethanol (TDE) diluted in PBS 1X, at room temperature, under agitation and protected from light. Samples were then preserved in 97% TDE at 4°C until observation under the microscope.

Each sample was placed in a POC chamber system (Pecon, Erbach, Germany) and observed under an inverted Yokogawa Spinning Disk confocal microscope (Carl Zeiss, Jena, Germany) with 10x/0.3 and 20x/0.4 long distance objectives. Full thickness 3D constructions were obtained from optical sections (z-stacks) acquired with the use of the ZEN acquisition software (Carl Zeiss, Jena, Germany).

### 2.6 Dissociation of cocultures

For cytometry of 3D cocultures, and transcriptomic analysis, coculture samples were dissociated using the Whole Skin Dissociation Kit, human (Miltenyi, Bergisch Gladbach, Germany) without enzyme P. Epidermis and dermis from a single skin coculture were mechanically separated from each other and processed separately when relevant.

### 2.7 Cytometry and fluorescence-activated cell sorting

Verification of B-cells activation was performed on a CytoFLEX (Beckman Coulter, Indianapolis, IN, USA) on cells stained with anti-CD25-PE (Biolegend, San Diego, CA, USA), anti-CD71-APC (Biolegend, San Diego, CA, USA) and LIVE/DEAD Fixable Aqua stain (ThermoFisher Scientific, Waltham, MA, USA).

B-cells phenotyping and FACS sorting were performed on a BD FACSAria™ III Cell Sorter (BD Biosciences), with a panel of antibodies composed of 7-AAD viability staining solution, anti-CD90-APC, anti-CD24-BV510, anti-CD45-AF488, & anti-IgD-PE from Biolegend (San Diego, CA, USA), and anti-CD19-PC7, anti-IgM-Pb & anti-CD38-ECD from Beckman Coulter (Indianapolis, IN, USA).

### 2.8 RNA extraction

Fibroblasts from 2D and 3D cocultures were processed for extraction of total RNAs using the NucleoSpin^®^ RNA Mini kit (Macherey-Nagel, Düren, Germany) as per the manufacturer’s instructions.

### 2.9 RT-qPCR

14,2 µl of each RNA sample were used for retro transcription into cDNA using the High-Capacity cDNA Reverse Transcription kit (ThermoFisher Scientific, Waltham, MA, USA). Quantitative real-time polymerase chain reactions (qPCRs) were performed on a selection of genes using Fast SYBR^®^ Green Master Mix (Life technologies, Waltham, MA, USA). Gene expression levels were calculated with the 2−ΔΔCt method, with GAPDH and HPRT1 as reference genes.

### 2.10 Cytokine analysis by Luminex

Human cytokines were determined in coculture supernatants using Human Magnetic Luminex Screening Assay (R&D Biosciences, Abingdon, UK) for MCP-1, IL-8, TNF-β and IL-6 in 2D cocultures.

### 2.11 Bulk RNAseq - on 2D cocultures

RNA quality (RIN) and concentration were measured on Bioanalyzer 2100 using RNA 6000 pico kit or RNA 6000 nano kit (Agilent Technologies, Santa Clara, CA, USA). 19 to 50 ng of RNA were used for library preparation with the QuantSeq 3’ mRNA-Seq Library Prep Kit FWD (Lexogen, Vienna, Austria) with UMI Second Strand Synthesis Module. Quality and concentration of cDNA libraries were measured on a Bioanalyzer 2100 (Agilent Technologies, Santa Clara, CA, USA), then all libraries were pooled equimolarly, and quality checked again. Sequencing was performed on a NovaSeq 6000 sequencing system (Illumina, San Diego, CA, USA) with 100 cycles for an objective of 20M reads per sample (using a single-end mode).

Control quality of sequencing data was realized using the fastp tool (v0.20.0). Reads were aligned on the human reference genome GRCh38 using STAR (v2.6) and Ensembl for gene annotation. UMI were processed with umitools (v0.5.4). The number of molecules per gene was calculated with FeatureCount (v1.6.0), and different programs were used for filtering data and checking quality along the process (qualimap (v2.2.1), fastp (v0.20.0), FastQC (v0.11.5) and MultiQC (v1.9)). The DESeq2 R bioconductor package (v1.30.1) was used for Differential Gene Expression, with the cut-off padj < 0.05.

### 2.12 BulkRNAseq - on 3D cocultures

RNA quality (RIN) and concentration were measured on Bioanalyzer 2100 using the RNA 6000 pico kit (Agilent Technologies, Santa Clara, CA, USA). About 100 ng of each sample were used for library preparations with the following kits: NEXTflex Poly(A) Beads 2.0. for isolation of mRNAs, NEXTflex Rapid Directional RNA-seq Kit 2.0 for cDNA generation and library construction, and NEXTFlex Unique Dual Index Barcodes - Set A for indexation (PerkinElmer, Shelton, CT, USA).

cDNA libraries were analyzed for length and concentration on Bioanalyzer 2100 with the DNA 1000 kit (Agilent Technologies, Santa Clara, CA, USA) and on Qubitflex 4 with the Qubit dsDNA BR assay kit (ThermoFisher Scientific, Waltham, MA, USA) respectively. Sequencing was performed on a NovaSeq 6000Dx sequencing system (Illumina, San Diego, CA, USA) in a 2x75bp configuration (using paired-end reads).

Demultiplexing of sequencing data was performed with bcl2fastq (v2.19.1.403), then adapters were removed with trimmomatic (v0.39). Reads were aligned on the human reference genome GRCh38 using the STAR aligner (v2.7.3a) and RSEM (v1.3.1) free softwares programs. Differential analysis was performed with the R package DESeq2 (v1.38.3).

### 2.13 Single-cell RNA-seq

Single-cell library preparation was realized using Chromium Next GEM Single Cell 5’ Kit v2 and Chromium Next GEM Chip K Single Cell Kit (10X Genomics, Pleasanton, CA, USA) as per the manufacturer’s instructions. Sequencing was performed on a NovaSeq 6000 sequencing system (Illumina, San Diego, CA, USA) using a 100bp chemistry (paired-end mode).

CellRanger analysis pipelines (v.7.0.1; 10X Genomics, Pleasanton, CA, USA) were used for the processing of single-cell RNA-seq data. In short, cellranger mkfast was used for data demultiplexing, then reads were aligned on the human reference genome GRCh38, filtered, and counted with cellranger count, and all samples were aggregated using cellranger aggr.

The Seurat package (v4.3.0) was operated on R (R4.2.2 2022–31-10) for quality filtering, clustering of cells and initial differential expression. Clustering was performed with or without BCR genes as previously described (13).

Cells with less than 200 expressed genes or more than 6947 expressed genes, dead cells with more than 10% of mitochondrial genes, and doublet cells (using scDblFinder tool) were excluded from further analysis. Gene expression of remaining cells was normalized with the LogNormalize method. JackstrawPlot and ElbowPlot allowed the selection of 30 principal components.

Unsupervised clustering of cells was realized with the k-NN method (k = 20) and Louvain algorithm (resolution = 0.8; FindClusters tool) and allowed the identification of 19 clusters visualized on a UMAP projection. Clusters were manually annotated based on gene markers representative of expected cell types, and differentially expressed genes were identified for each cluster (|FC| > 1.5; padj < 0.05). Follow-up analysis and comparisons between clusters and conditions were realized with the Loupebrowser software (10X Genomics, Pleasanton, CA, USA) using the UMAP mapping and the clustering from Seurat. Differential expression analysis was performed with a minimum of 200 cells per group.

### 2.14 Data processing and statistics

Confocal images were analyzed with the 3D Imaris software (Oxford Instruments, Abingdon, UK). Bulk RNA-seq data was analyzed and formatted using the UseGalaxy platform. Formatting and statistics were performed using the Prism software (GraphPad, San Diego, CA, USA). All transcriptomic data is available at the SRA repository (accession number PRJNA1110840).

## 3 Results

### 3.1 B-cells infiltrate the 3-dimensional healthy skin model

We developed a model of coculture between a 3-dimensional healthy skin model and B-cells from healthy donors or patients with SSc. B-cells were stimulated or not with a mix of antibodies against BCR, ligand of the CD40 receptor (CD40L) and CpG oligodeoxynucleotides (CpG ODN) during 24h to 48h prior to coculture.

We first assessed the intensity, kinetics and depth of infiltration in the skin by healthy activated B-cells (HC A B-cells), after 2 to 6 days of coculture. For each timepoint, the number of infiltrated cells and the depth of infiltration were measured (**Supplementary Figures 1**, **2A**). The infiltration of B-cells occurred as early as 2 days of coculture (**Supplementary Figure 2A**), and spread into the skin between 20 and 30 µm of depth (**Supplementary Figure 2B**). Infiltration increased between 2 and 3 days of coculture and then remained stable (**Supplementary Figure 2A**). The depth of infiltration was significantly increased at 2 and 4 days of coculture when compared to the other timepoints, although the differences were slight (**Supplementary Figure 2B**). Following these results, we thus chose a duration of 4 days for further 3D cocultures, as it allowed a significant infiltration of B-cells and was compatible with technical constraints.

### 3.2 Activated B-cells from SSc patients and healthy controls infiltrate more the 3-dimensional healthy skin model than non-activated B-cells

We next investigated whether the infiltration in the skin model was influenced by the origin (SSc patients or healthy subjects) or the activation state of B-cells.

**Figure 1:**
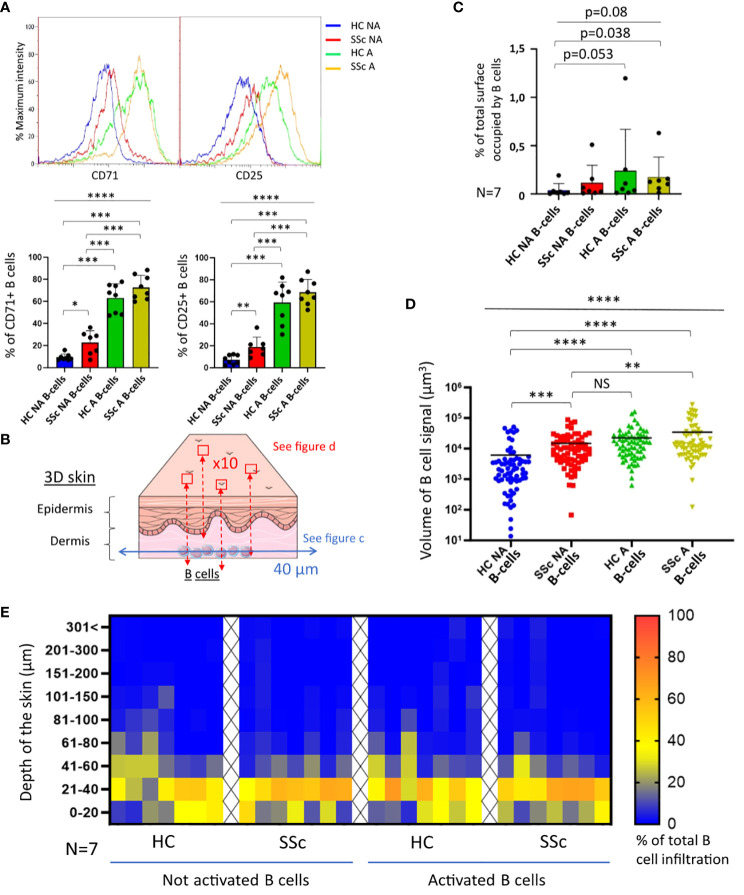
Infiltration of B-cells from healthy and SSc patients in 3D healthy skin. **(A)** State of proliferation and activation of B-cells before coculture, measured respectively via CD71 and CD25 markers by cytometry (N=8 patients and healthy subjects). **(B)** Illustration of the strategy used to collect confocal microscopy images after 4 days of coculture. The results of image analysis are presented in **(C)** with the quantification of B-cell signal at 40 µm (% of total surface occupied by B-cell signal normalized with the number of viable B-cells used; N=7), and in **(D)** with the volume occupied by B-cell signal quantified in the whole thickness of the skin (N=7 cocultures per condition; 10 images per patients and healthy subjects). **(E)** Repartition of B-cell infiltration represented by the % of total B-cell infiltration in the different levels of skin depth. Kruskal-Wallis non-parametric test (bar) and Mann-Whitney unpaired t-test (bracket). Mean +/- SD. NS = not significant; *p<=0.05; **p<=0.01; ***p<=0.001; ****p<=0.0001. HC NA B-cells, non-activated B-cells from healthy subjects; SSc NA B-cells, non-activated B-cells from SSc patients; HC A B-cells, activated B-cells from healthy subjects; SSc NA B-cells, non-activated B-cells from SSc patients. Panel **(B)** was partly generated using Servier Medical Art, provided by Servier, licensed under a Creative Commons Attribution 3.0 unported license.

The first step was the evaluation of B-cells activation state prior to coculture. At basal state, CD25 and CD71 expression on B-cells were significantly higher in patients with SSc when compared to healthy subjects, suggesting a higher basal activation and proliferation (**Figure 1A**). For both markers, the proportion of positive B-cells strongly and significantly increased after stimulation, from 20% to more than 60% both in SSc patients and healthy subjects, with no significant difference between these 2 populations (**Figure 1A**).

We then cocultured activated or non-activated B-cells with the skin model. After 4 days of coculture, the infiltration of B-cells in 3D skin was characterized by confocal microscopy. For each coculture sample, two different image captures were taken (**Figure 1B** and **Supplementary Figure 1**): transversal images taken at 40 µm from the base of the skin, and compilation of images taken at regular intervals of depth of 2.4 µm (z-stack) allowing the full-thickness 3-dimensional representation of the skin. B-cells infiltration was observed for all conditions (HC/SSc/activated or not B-cells), on transversal images taken at 40 µm from the base of the skin (**Figure 1C**). We observed a trend on a weaker infiltration at 40 µm by healthy non-activated B-cells (HC NA B-cells) when compared to the other conditions. On a full-thickness analysis of cocultured skins, we showed that the activation of B-cells was associated with a significant increase of their infiltration, independently of the origin of the cells (SSc patients or healthy subjects) (**Figure 1D**). By contrast, we observed that the infiltration was similar between SSc non-activated B-cells (SSc NA B-cells) and HC A B-cells (**Figure 1D**). Most B-cells were infiltrated within the first 60 µm of the dermis in all conditions (**Figure 1E**).

### 3.3 B-cell homeostasis is modified after 3D cocultures

Cytometry analysis of healthy and SSc B-cells before and after coculture allowed the analysis of the repartition of transitional, mature naïve, unswitched and switched memory B-cells, and plasmablasts (**Supplementary Figure 3**).

**Figure 2:**
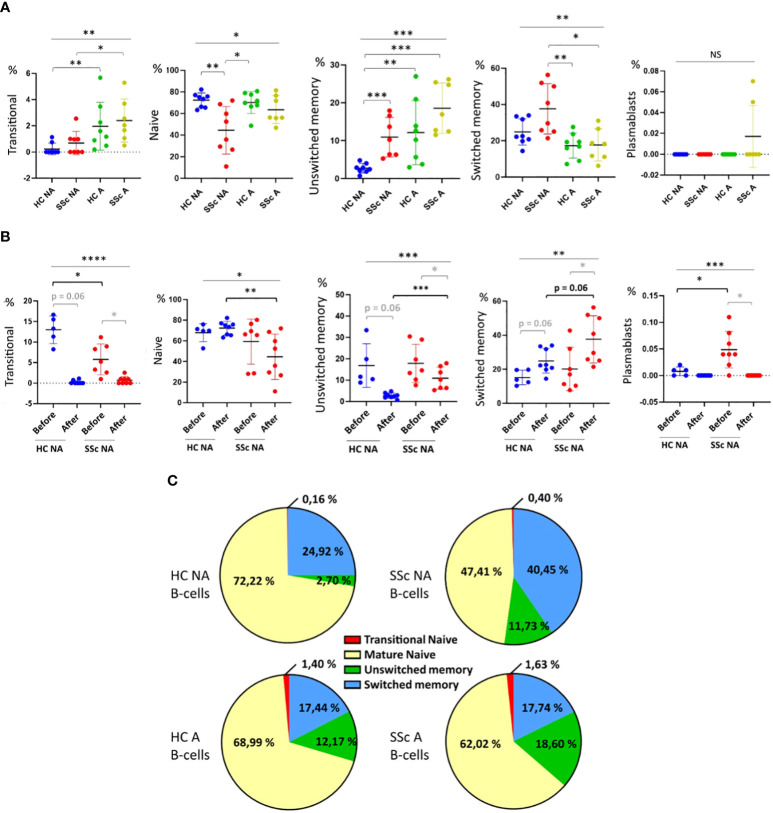
Characterization of B-cells sub-populations before and after 3D cocultures. Percentages of transitional (IgD^+^ CD24^high^ CD38^high^), naïve (IgD^+^), unswitched memory (IgM^+^ IgD^low^), switched memory (IgM^-^ IgD^-^) B-cells and plasmablasts (CD24^-^ CD38^+^) among CD19^+^ cells. **(A)** after 4 days of coculture with healthy or SSc B-cells, activated or not, and **(B)** before and after 4 days of coculture with healthy or SSc non-activated B-cells. **(C)** Repartition of B-cells sub-populations after coculture depending on B-cell origin and activation state. Kruskal-Wallis non-parametric test (black bar) and Mann-Whitney unpaired t-test (black bracket) and Wilcoxon paired t-test (grey bracket). Mean +/- SD. NS = not significant; * p<=0.05; ** p<=0.01; *** p<=0.001. HC NA B-cells, non-activated B-cells from healthy subjects; SSc NA B-cells, non-activated B-cells from SSc patients; HC A B-cells, activated B-cells from healthy subjects; SSc NA B-cells, non-activated B-cells from SSc patients.

After 4 days of coculture, we observed that the B-cell infiltrates were mainly composed of naïve IgD^+^, unswitched memory IgM^+^ IgD^low^, and switched memory IgM^-^ IgD^-^ cells in all conditions (SSc and healthy B-cells, with or without activation). In the SSc NA B-cells condition, the proportion of switched memory B-cells among infiltrated CD19^+^ cells was higher and the proportion of naïve B-cells lower than in other conditions, (**Figures 2A, C**). The proportion of unswitched memory B-cells was lower in the HC NA B-cells condition when compared to the other conditions. Activation of B-cells increased the proportion of transitional B-cells among the infiltrated B-cells after coculture both in SSc patients and healthy subjects. Only very few plasmablasts were identified after coculture (**Figures 2A, C**).

We then assessed the modification of B-cell population distribution between before and after coculture. However, on a technical point, we observed a decreased expression of all surface markers on freshly activated B-cells before coculture, precluding the identification of B-cell subpopulations before coculture for activated HC and SSc B-cells (**Supplementary Figure 3B**). Thus, the comparison of B-cell subpopulations distributions before and after coculture was only possible for NA B-cells conditions (**Figure 2B**). In the SSc NA B-cells condition, the proportion of transitional, naïve and unswitched memory B-cells as well as plasmablasts decreased after coculture, while the proportions of switched memory B-cells increased. (**Figure 2B**). After coculture and for HC NA B-cells, we also observed a decrease in the proportions of transitional B-cells and plasmablasts while the proportion of naïve B-cells was similar and the proportion of switched memory B-cells higher, when compared to the condition “before coculture”. The increase in switched memory B-cell skin infiltration was higher for SSc NA B-cells than the one observed with HC B-cells (+20 vs +10% of total B-cells, respectively) (**Figure 2B**). The proportion of infiltrated unswitched memory B-cells decreased in both SSc and HC NA B-cells condition compared to the before coculture condition (p=0.016 and p=0.06, respectively).

**Figure 3:**
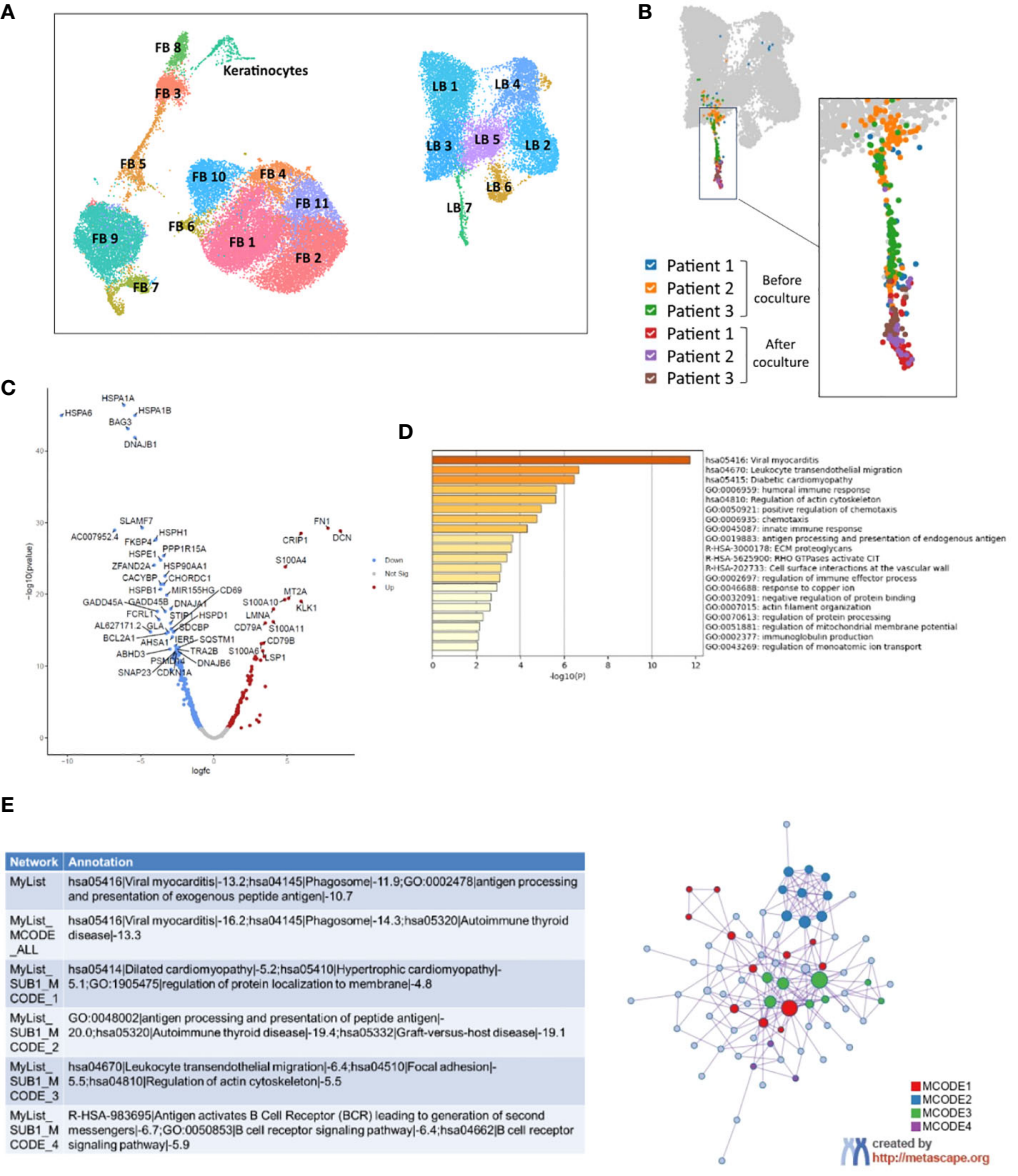
Analysis by single-cell RNAseq of activated B-cells from patients with systemic sclerosis before and after 5 days of coculture with healthy 3D skin. **(A)** UMAP visualization of analyzed cells from three 3D skin models alone, control SSc A B-cells from three SSc patients and three cocultures between 3D healthy skin models and SSc A B-cells. **(B)** Focus on LB 7 cluster, which includes all B-cells after coculture. **(C)** Volcano plot representing the most significant differentially expressed genes in B-cells of LB 7 cluster after coculture compared to before coculture (threshold: padj > 0.05). **(D)** Enrichment in GOTERM and molecular pathways and **(E)** Protein-protein interactions network and MCODE components identified after analysis by Metascape of genes overexpressed in c. FB, Fibroblast, LB, B-cell.

Because cytometry could not provide robust information about the impact of coculture in the conditions with activated B-cells, we completed the experiments with a single-cell RNA sequencing approach in the conditions with SSc activated B-cells (**Figure 3**).

### 3.4 Single-cell RNA-seq analysis reveals a marked activated profile in infiltrated B-cells from patients with systemic sclerosis.

Skin models alone (n=3), SSc activated B-cells (SSc A B-cells) before coculture (circulating) and after 3D coculture (infiltrated) (n=3) were analyzed. Bioinformatic analysis of the whole dataset identified 19 clusters composed of B-cells, fibroblasts and keratinocytes (**Figure 3A**; **Supplementary Figure 4**).

Among B-cell clusters, the “LB7” cluster contained all infiltrated B-cells from the coculture conditions (**Figure 3B**). Inside this cluster, B-cells before and after coculture were distinctly localized on a UMAP representation, suggesting the expression of a different transcriptomic profile (**Figure 3B**). A differential analysis of B-cells from cluster LB7 identified 313 differentially expressed genes (DEGs) between before and after coculture (|FC| > 1.5, padj < 0.05), among which 100 genes were upregulated after coculture and 213 genes downregulated (**Figure 3C**). An enrichment in GO terms, KEGG and REACTOME pathways for B-cell genes overexpressed after coculture was conducted using the DAVID and METASCAPE tools (with Benjamini-Hochberg correction method). We observed a significant enrichment in terms associated with viral myocarditis (KEGG Pathway hsa05416) and leukocyte transendothelial migration (KEGG Pathway hsa04670), illustrated by the positive regulation of several genes from the cytoskeleton (actin, coronin 1A, RAC1…) (**Figure 3D**, **Supplementary Figures 5A, C**). An enrichment in cyclooxygenase genes (KEGG Pathway hsa05415 Diabetic cardiomyopathy) favored a further activated profile of B-cells after coculture (**Figure 3D**, **Supplementary Figure 5B**). GO terms “B-cell receptor signaling pathway” and “immune response” were also present in the enrichment, with a prevalence of DEGs associated with immunoglobulins and major histocompatibility complex (**Figure 3D**, **Supplementary Figures 5D–F**). These cellular functions and molecular pathways were also found in protein-protein interactions networks (**Figure 3E**). Several molecules from the S100 family such as S100A4 belonged to the list of enriched genes, and we also observed the overexpression of CXCR4, associated with tissue homing by B-cells (14), by infiltrated B-cells in the LB7 cluster.

While we observed a clear modification of the transcriptome of B-cells analyzed in single cell between before and after coculture, there was only one gene (ANGPTL4; padj = 0.01) upregulated in fibroblasts cocultivated with SSc A B-cells compared to control skin. By contrast, we found 43 DEGs for keratinocytes between before and after coculture, among which GOterms related to keratinization and differentiation were enriched (**Supplementary Figure 6**).

As performed in the literature (13), we re-runed the clustering and all downstream analysis on our single cell RNAseq data while removing genes coding for the BCR. Our main results remained unchanged, but the few modifications in are visible in **Supplementary Figure 7**.

**Figure 4:**
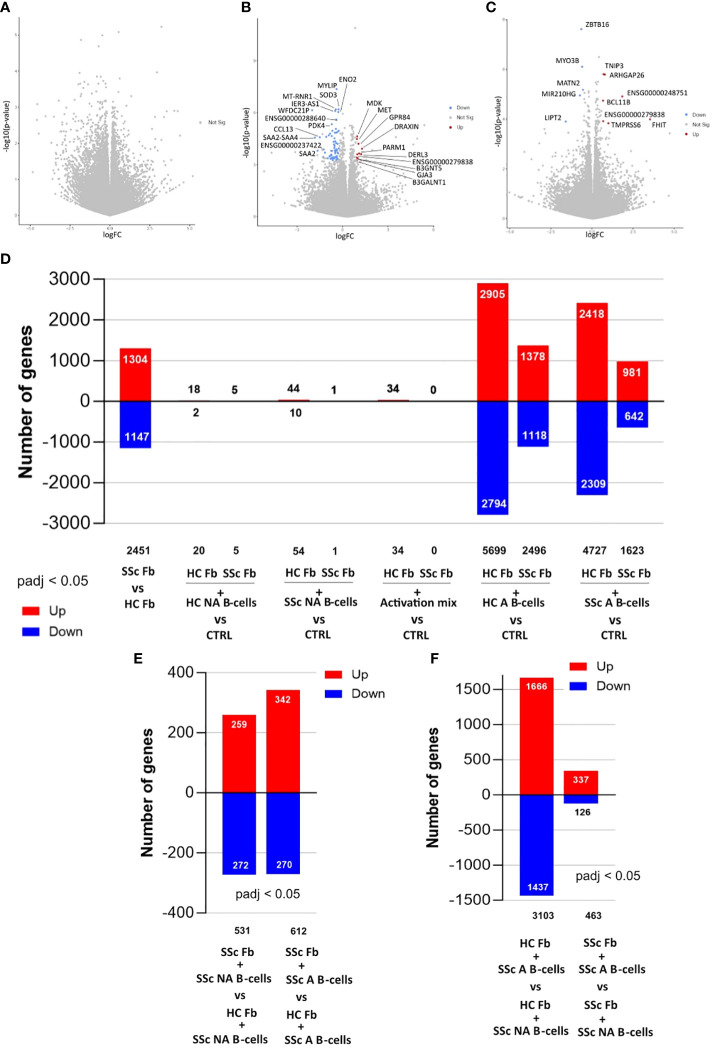
Differentially expressed genes in fibroblasts from 3D (4 days) and 2D (2 days) cocultures with B-cells analyzed with bulk-RNAseq. Volcano plots of **(A)** Comparison between healthy fibroblasts cocultured in 3D with healthy non-activated B-cells (HC NA B-cells) vs healthy control fibroblasts alone **(B)** Comparison between healthy fibroblasts cocultured in 3D with SSc non-activated B-cells (SSc NA B-cells) vs healthy control fibroblasts alone **(C)** Comparison between healthy fibroblasts cocultured in 3D with SSc NA B-cells vs healthy fibroblasts cocultured in 3D with HC NA B-cells. Significance thresholds: |FC| > 1.5 and padj < 0.05. Number of genes differentially expressed in fibroblasts with **(D)** Comparison between SSc (SSc Fb) and HC (HC Fb) fibroblasts; Comparison between HC Fb or SSc Fb cocultured in 2D with HC NA B-cells vs Fb alone, HC or SSC, (CTRL); Comparison between HC Fb or SSc Fb cocultured in 2D with SSc NA B-cells vs HC Fb or SSc Fb (=CTRL); Comparison between HC Fb or SSc Fb cocultured in 2D with activation mix (anti-BCR, CD40L/anti-histidine, CpG) vs HC Fb or SSc Fb (=CTRL); Comparison between HC Fb or SSc Fb cocultured in 2D with healthy activated B-cells (HC A B-cells) vs HC Fb or SSc Fb (=CTRL); Comparison between HC Fb or SSc Fb cocultured in 2D with SSc A B-cells vs HC Fb or SSc Fb (=CTRL); **(E)** Comparison between HC Fb cocultured in 2D with SSc NA B-cells or SSc A B-cells vs SSc Fb cocultured in 2D with SSc NA B-cells or SSc A B-cells. **(F)** Comparison between HC Fb cocultured in 2D with SSc NA B-cells vs SSc A B-cells; Comparison between SSc Fb cocultured in 2D with SSc NA B-cells vs SSc A B-cells. Significance thresholds: padj < 0.05. HC, Healthy control; NA, non-activated; A, activated; Fb, Fibroblasts; SSc, Systemic sclerosis.

Thus, to further explore the effect and mechanisms of B-cells/skin interactions at the transcriptional level, fibroblasts from 3D cocultures between healthy skin models and B-cells (activated or not) from healthy subjects (N=8) or SSc patients (N=8) were analyzed by bulk RNA-sequencing (**Figure 4**).

### 3.5 The transcriptome of fibroblasts is modified by the cocultures with B-cells depending on the origin and activation status of the cells

3D cocultures with HC NA B-cells did not impact the transcriptome of fibroblasts from the healthy 3D skin model compared to control fibroblasts (**Figure 4A**). SSc NA B-cells modified the bulk transcriptome of fibroblasts in 3D coculture compared to control fibroblasts (**Figure 4B**), thus with a differential impact on the fibroblast transcriptome between these 2 conditions (12 DEGs, see **Figure 4C**). For the conditions with activated B-cells, we observed that the transcriptome of fibroblasts after coculture included many genes specific for B-cells (CD20, Immunoglobulins…) suggesting a contamination of fibroblasts by B-cells, despite the FACS sorting. This precluded any firm conclusion concerning the impact of activated B-cells on bulk transcriptome of fibroblasts in the 3D model.

**Figure 5:**
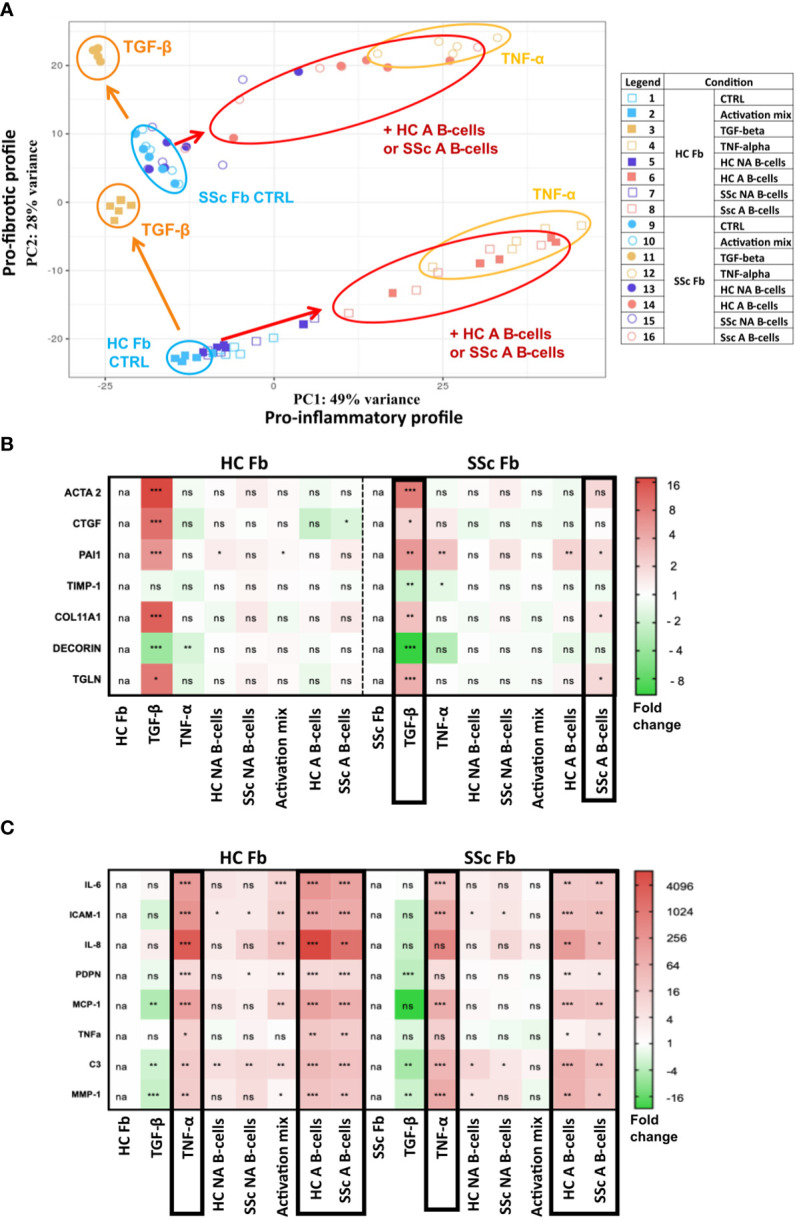
Pro-fibrotic and pro-inflammatory profiles of healthy control (n=1) and systemic sclerosis fibroblasts (n=1) after 2D coculture with activated and non-activated B-cells from healthy controls (n=5) and patients with systemic sclerosis (n=5). **(A)** Principal component analysis of bulk RNAseq data. The different conditions are displayed on the right. Heatmaps represent the relative expression of **(B)** pro-fibrotic and **(C)** pro-inflammatory genes measured by qPCR according to the different culture conditions. Framed results highlight conditions with similar profiles. Unpaired t-test (Mann-Whitney). *p<=0.05 **p<=0.01 ***p<=0.001. NS, not significant; HC NA B-cells, non-activated B-cells from healthy subjects; SSc NA B-cells, non-activated B-cells from SSc patients; HC A B-cells, activated B-cells from healthy subjects; SSc NA B-cells, non-activated B-cells from SSc patients.

Thus, to gather data on the impact of activated B-cells on fibroblasts, we decided to complete this study with 2-dimensional cocultures between healthy or SSc fibroblasts and healthy or SSc B-cells, activated or not. The analysis of fibroblasts using bulk RNA-sequencing showed an intrinsic difference of 2,451 DEGs between HC and SSc fibroblasts at basal state (**Figures 4D**, **5A**). This difference between SSc and HC fibroblasts transcriptome were also observed after coculture with B-cells (**Figure 4E**).

Overall, activated B-cells had a more important impact on both healthy and SSc fibroblasts compared to non-activated B-cells in 2D cocultures (1,500 to 5,500 DEGs after B-cells activation versus less than 50 DEGs without activation) (**Figure 4D**). Activated B-cells had a stronger impact on healthy fibroblasts (4,000 DEGs identified, in average, in HC fibroblasts cocultured with healthy or SSc B-cells versus control HC fibroblasts alone) than on SSc fibroblasts (1,500 to 2,500 DEGs in average, in SSc fibroblasts cocultured with healthy or SSc B-cells versus control SSc fibroblasts alone) (**Figure 4D**). The comparison of fibroblasts in different coculture conditions confirmed that B-cells have a different impact on the transcriptome of fibroblasts depending on B-cells activation state (**Figure 4F**), independently from the origin of B-cells (data not shown).

In a principal component analysis of fibroblast transcriptomic profiles for all coculture conditions (based on the 500 most variable genes) we observed the two components obtained were respectively associated with fibroblasts stimulated with the pro-inflammatory cytokine TNF-α (component 1) or the pro-fibrotic cytokine TGF-β (component 2) (**Figure 5A**). We observed that SSc fibroblasts showed a profile close to the one of healthy fibroblasts when stimulated with TGF-β (**Figure 5A**). The coculture with activated B-cells changed both SSc and healthy fibroblasts expression profile toward a pro-inflammatory transcriptomic profile similar to TNF-α stimulation. However, healthy fibroblasts stimulated with TNF-α or activated B-cells, and SSc fibroblasts stimulated with TNF-α or activated B-cells remained separated on the PCA. Indeed, the pro-fibrotic shift observed on component 2 between healthy and SSc fibroblasts at basal state, remained when both fibroblasts were either stimulated with TNF or cocultured with activated B-cells (**Figure 5A**). This was further confirmed by the expression of several pro-inflammatory and pro-fibrotic genes measured in cocultured fibroblasts by qPCR (**Figures 5B, C**).

The analysis of the 200 most significantly overexpressed genes in SSc fibroblasts in 2D cocultures with SSc A B-cells, compared to SSc fibroblasts alone, showed an enrichment in GO terms and KEGG pathways associated with cytokines signaling (R-HSA-1280215, GO:0071345, GO:0001819) (**Supplementary Figures 8A, G, H**) and a pro-inflammatory response (**Supplementary Figure 8F**). These data are illustrated by terms associated with NF-kB signaling (GO:0043122 regulation of canonical NF-kappa-B signal transduction, R-HAS-5668541 TNFR2 non-canonical NF-kappaB signal transduction) (**Supplementary Figure 8A**), as well as by the deregulation of several transcriptional factors (**Supplementary Figure 8B**) and multiple genes that are involved in the response to TNF-α (**Supplementary Figure 8C**), IL-17 (**Supplementary Figure 8D**), and the NOD-like receptors pathway (**Supplementary Figure 8E**). When comparing the transcriptome of SSc fibroblasts after coculture with activated vs non-activated SSc B-cells, a similar pro-inflammatory profile was observed based on GO terms (**Supplementary Figures 9A, D, E**) and deregulated transcription factors (**Supplementary Figure 9B**). Strong TNF-α and INF signaling signatures were also found among the gene enrichment (R-HSA-877300 Interferon gamma signaling, GO:0034341 response to type II interferon) (**Supplementary Figures 9A, F**) and protein-protein interactions network analyses (**Supplementary Figure 9C**).

To summarize, 3D and 2D coculture experiments highlighted complementary findings about the impact of B-cells on the transcriptomic profile of fibroblasts both in the healthy and SSc conditions. In 3D cocultures, fibroblasts were differentially impacted by the coculture with healthy vs SSc B-cells in the NA B-cells conditions. In 2D cocultures, the origin of fibroblasts and the state of activation of B-cells, but not the origin of B-cells, had a differential effect on healthy and SSc fibroblasts. Overall, the interaction with activated B-cells induced a pro-inflammatory profile with an activation of the TNF pathways in both healthy and SSc fibroblasts, and in a lesser extent a profibrotic profile.

Following the analysis of the transcriptomic data obtained for fibroblasts in 2D cocultures with B-cells, we decided to further explore the role of cytokines in B-cell/fibroblast interactions.

### 3.6 Blocking TNFs inhibit the pro-inflammatory profile of fibroblasts induced by the 2D coculture with B-cells

**Figure 6:**
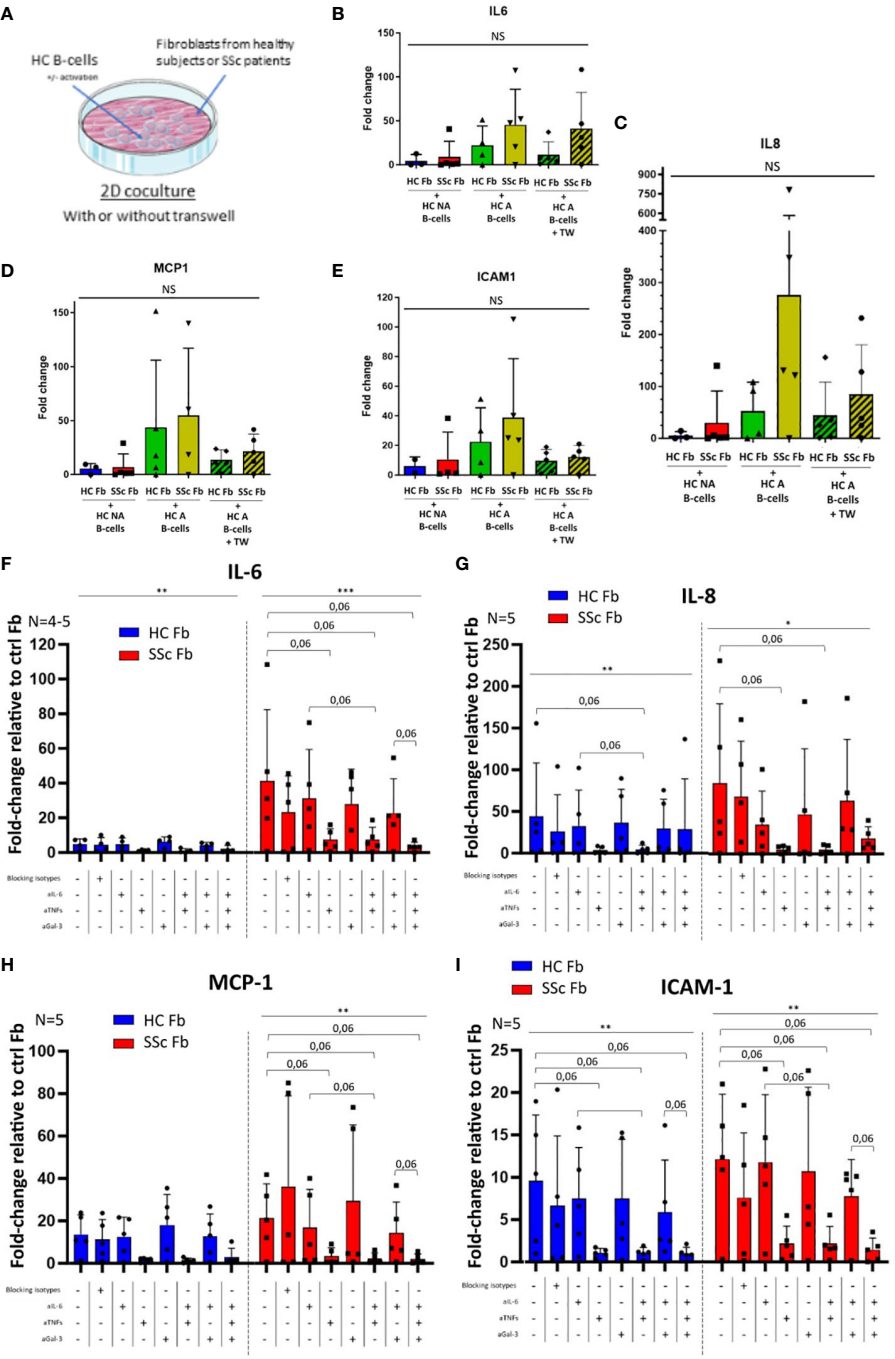
Expression of pro-inflammatory markers in healthy or SSc fibroblasts after 2 days 2D cocultures with healthy B-cells with or without blocking. **(A)** Experimental plan for 2D cocultures. N = 1 healthy subject for B-cells vs 4 to 5 healthy subjects for HC Fb and 4 to 5 SSc patients for SSc Fb, for all figures. Without blocking: Gene expression is measured by qPCR, normalized with GAPDH and HPRT1, in the different conditions expressed in fold-change relative to control fibroblasts: IL-6 **(B)**, IL8 **(C)**, MCP-1 **(D)** and ICAM1 **(E)**. Statistical analysis with Kruskal-Wallis non-parametric test (bar). With blocking: HC B-cells were activated and separated from HC or SSc fibroblasts with a transwell in the different conditions. Genes expression is measured by qPCR, normalized with GAPDH and HPRT1 expressed in fold-change relative to control fibroblasts: IL-6 **(F)**, IL8 **(G)**, MCP-1 **(H)** and ICAM1 **(I)**. Statistical analysis with Friedman non-parametric paired test (bar; separate for HC Fb and SSc Fb conditions) and Wilcoxon paired t-test (bracket). Mean +/- SD. * p<=0.05; ** p<=0.01; *** p<=0.001; **** p<=0.0001. NS, not significant; HC NA B-cells, non-activated B-cells from healthy subjects; SSc NA B-cells, non-activated B-cells from SSc patients; HC A B-cells, activated B-cells from healthy subjects; SSc NA B-cells, non-activated B-cells from SSc patients; HC Fb, control fibroblasts from healthy subjects; SSc Fb, control fibroblasts from SSc patients; aIL-6, anti-IL-6 inhibition; aTNFs, anti-TNF-α and anti-TNF-β inhibition; aGal-3, anti-galectin-3 inhibition.

We chose a 2D coculture setting of 2 days between healthy B-cells (activated or not) and healthy or SSc fibroblasts, with or without transwell for the HC A B-cell conditions. To study how the inhibition of specific cytokines could impact the interactions between the two cell types, we measured the expression of different pro-inflammatory genes in fibroblasts using qPCR (**Figure 6**).

Without any blocking, in 2D cocultures, the expression of IL-6, IL-8, ICAM1 and MCP-1 in healthy or SSc fibroblasts in presence of HC NA or HC A B-cells did not vary in a significant manner between conditions (**Figures 6B–E**). Of note, we observed a non-significant increase of all gene expression for cocultures with activated B-cell compared to non-activated B-cell conditions, and a non-significant decrease of MCP-1 and ICAM1 for conditions with transwell compared to without transwell.

We chose to inhibit IL-6, TNF-α/-β and galectin-3 in various combinations, in 2D cocultures between healthy activated B-cells and healthy or SSc fibroblasts separated by transwells to forbid direct contact between cell types (**Figure 6A**). When TNF-α and TNF-β were inhibited, we observed a trend toward a decrease in IL-6, IL-8, MCP-1 and ICAM1 gene expression in fibroblasts after 48h of coculture (p=0.06) (**Figures 6F–I**).

## 4 Discussion

The main results of our study focusing on the interactions between B-cells from patients with SSc and a 3D model of skin are the following: a) circulating B-cells are able to infiltrate a 3D healthy skin model, b) the amount but not the depth of infiltration are higher with B-cells from SSc patients and with activated B-cells, c) B-cell infiltrates include a majority of naïve and memory cells, and subpopulations frequencies vary depending on B-cells origin (HC or SSc) and activation state, d) infiltrated B-cells from patients with SSc show an activated profile and an overexpression of immunoglobulin genes, e) activated B-cells have an impact on the transcriptomic profile of both healthy and SSc fibroblasts, toward a pro-inflammatory (TNF and IL-17 signaling) and interferon signatures, with a key role of the TNF pathway.

The first result of our study is that B-cells can infiltrate a 3D skin model. The use of this 3D skin model allowed us to modelize the infiltration of B-cells from blood into the skin, mimicking what is observed *in vivo*, as attested by several detections of B-cell populations in both healthy skin (15–17) and lesional skin from SSc patients (5–7). To our knowledge, the only other example of 3D coculture for the study of skin/immunity interactions is the infiltration of T cells in a reconstituted healthy skin model in the context of psoriasis (18), where the authors show that chemokines produced by the skin may attract T-cells. In our study, SSc B-cells expressed CXCR4 both before coculture and after infiltrating the 3D skin model. The CXCR4/CXCL12 axis seems particularly associated with B-cells infiltration in single-cell analysis of SSc skin biopsies (7) and is also involved in cutaneous B-cell infiltration in bullous pemphigoid (14).

Secondly, we observed an increased infiltration (i.e., a higher number of infiltrated B-cells in 3D skin) of both healthy and SSc activated B-cells when compared to non-activated B-cells. This observation was clearly visible when quantifying infiltration on the whole thickness of cocultured skin (**Figure 1D**) rather than limiting the analysis at a depth of 40µm (**Figure 1C**). Analyzing the infiltration on the whole thickness appears as more informative and valid, the method used for image analysis is also more precise, and the number of images analyzed per coculture is higher. We showed that *in vitro* activation of B-cells with both T-dependent (CD40L, BCR stimulation) and T-independent (CpG) stimuli led to a higher B-cell expression of CD25 and the proliferation marker CD71. Activation may induce a higher capacity of B-cells to infiltrate, by affecting the expression of chemokine receptors, adhesion molecules or ECM remodeling factors. Interestingly, the infiltration by SSc NA B-cells was similar to the infiltration by HC A B-cells. To explain this result, we showed that SSc NA B-cells had at basal state a more activated phenotype than HC NA B-cells (overexpression of CD25 and CD71). This result is in line with our previous study showing that SSc peripheral B-cells present a high expression of different activation markers (CD19, CD80, CD95, HLA-DR) (19) and particularly among memory B-cells (20). This basal activated state of SSc B-cells probably explains why they infiltrate more the 3D skin model than their HC NA B-cells counterpart.

After analyzing the number of infiltrated B-cells, we next showed that the depth of infiltration by B-cells was heterogenous within and between the different conditions and was not modified by their origin or state of activation after 4 days of coculture. We have no firm explanations for this result. We can hypothesize that 4 days of coculture could not be sufficient to observe a difference in depth of infiltration between the conditions. Moreover, individual variations in age, or in disease characteristics for SSc patients could have an impact and partly explain the heterogeneity.

Thirdly, we showed that B-cells infiltrating the 3D skin model were composed predominantly of mature naïve, unswitched memory and switched memory cells, as identified in cytometry experiments. To our opinion, these results are important since no detailed analysis of B-cell subtypes found in the tissues of SSc patients is available in the literature. In the SSc NA B-cells condition, we showed that 1. the proportion of switched memory B-cells among infiltrated CD19^+^ cells was higher and of naïve B-cells lower than in other conditions 2. the proportion of switched memory B-cells increased between before and after coculture, while the proportion of transitional, naïve and unswitched memory B-cells as well as plasmablasts decreased. Interestingly, the different subtypes of circulating B-cells are known to vary between SSc patients and healthy subjects (19–24): usually, circulating memory B-cells are decreased but activated in SSc, while naïve B-cells are increased. To explain these results, we could thus hypothesize that circulating activated memory B-cells from patients with SSc infiltrate the skin, resulting in an increased proportion of these cells in the skin and a decreased proportion in the blood. This raises the question of the ability of memory B-cell to infiltrate more than other B-cells subtypes. A recent single-cell analysis of sorted B-cell from healthy human blood suggested a differential expression of genes associated with cell adhesion and cytoskeletal modifications between B-cell subtypes (25). Very few plasmablasts were observed among infiltrated B-cells in our study. Yet, plasmablasts seem to play an important role in SSc pathophysiology, although the presence of plasma cells *in vivo* in tissues of SSc patients appears to be heterogeneous between patients (20). The main hypotheses to explain why we do not observe a large amount of infiltrating plasmablasts after co-culture might be that 1. plasmablasts have lower infiltration capacity than other subpopulations 2. 4–5 days of coculture may not be enough to allow preexisting plasmablasts to fully infiltrate or for other infiltrated B-cells to differentiate into plasmablasts.

We were not able to analyze the modification of B-cell subtypes by cytometry to compare before and after coculture in the activated conditions. We thus analyzed by single cell the modifications of SSc A B-cells before and after coculture. Previous single-cell sequencing studies only focused on distinguishing B-cell and plasma cell populations in skin biopsies of SSc patients (7). In our study, the single-cell transcriptomic analysis of SSc activated B-cells in coculture with 3D skin showed an overactivation of SSc infiltrated B-cells compared to the same cells analyzed before coculture. This is illustrated by the overexpression of CD79a and CD79b, molecules involved in the transduction of BCR signal, and of CD81 whose co-expression with BAFFR and CD38 in transitional B-cells is associated with active systemic lupus erythematosus (26). Infiltrated B-cells also showed an upregulation of genes associated with antigen-presentation and immunoglobulin secretion properties. This could suggest a maturation process toward a profile of antibody-secreting cells (ASCs), as described in the joint of rheumatoid arthritis patients (27). We acknowledge that there is an apparent discrepancy between FACS (virtually no plasmablast) and single cell RNA-seq (ASC profile) data presented here on the matter of infiltrated B-cells identity. To explain this discrepancy, we hypothesize that 1. B-cells infiltrating the skin in cocultures could be engaged in maturation toward an ASC profile but are not terminally differentiated yet. In this case, this could explain why we found the overexpression of immunoglobulin genes which has not been translated to surface protein markers visible in cytometry. 2. the different length of coculture between single cell RNAseq (5 days) and cytometry (4 days) experiments could impact the maturation of B-cells in response to both stimulation and coculture. 3. we used different markers between single cell (Ig genes) and cytometry (CD19, CD24, CD38). The evaluation of intracellular immunoglobulin molecules expression by cytometry could be an interesting perspective to our analysis.

Cyclooxygenase (COX) genes were also present in the enrichment analysis of SSc infiltrated B-cells. These molecules are highly expressed by activated B-cells upon stimulation with CD40L and BCR engagement and are known for their involvement in antibody production and class switching (28, 29). Altogether our results suggest that B-cells infiltrating the tissues in SSc could be a local source of auto-antibody secretion. This deserves further study isolating infiltrated B-cells and assessing their ability to produce autoantibodies. Moreover, several molecules from the S100A family were found upregulated in infiltrated SSc A B-cells in single-cell. Among the top 5 upregulated genes, S100A4 is known for its implication in T lymphocyte chemoattraction and its amplification of fibroblasts activation via TGF-β in SSc pathology (30).

After having assessed the effect of coculture on B-cells, we then focused on their impact on fibroblasts. A single DEG was found, in single-cell analysis, between fibroblasts cocultivated or not with SSc A B-cells. This could be explained by a low proportion of fibroblasts in contact with B-cells in the 3D setting. To address this issue, we assessed the effects of B-cells on fibroblasts in 2D coculture, where the proportion of fibroblasts in contact with B-cells is much higher.

In 2D cocultures, we showed a limited impact of NA B-cells on healthy fibroblasts. In contrast, fibroblasts in 2D cocultures with activated B-cells had an important modification of their transcriptome compared to fibroblasts alone, with a higher effect on HC fibroblasts than SSc fibroblasts. We showed, with a global transcriptomic approach, that the profile induced by coculture with activated B-cells was close to the one induced by TNF-α stimulation, and involved the upregulation of several pro-inflammatory genes. Especially in the SSc A B-cells and SSc fibroblasts 2D cocultures setting, the genes upregulated in fibroblasts pointed toward inflammation and cytokine signaling, with a signature for interferon gamma, TNF-α and IL-17. The pro-inflammatory profile of fibroblasts was already present in the coculture with SSc NA B-cells, and further enhanced when SSc B-cells were activated. Such a pro-inflammatory profile has been observed in skin biopsies of localized scleroderma, including IFN signaling and HLA-associated genes upregulation (31).

In addition, we showed that the inhibition of TNF-α and TNF-β in 2D cocultures involving HC A B-cells led toward the downregulation of several pro-inflammatory cytokines in fibroblasts, comforting the hypothesis of TNF-α involvement in our model. Moreover, this cytokine is an important player in SSc. SSc fibroblasts are refractory to the anti-fibrotic effect that TNF-α exerts on healthy cells (32) and is involved in the proinflammatory profile observed in the lung fibrosis in a murine model of SSc with bleomycin (33). The different pro-inflammatory factors produced by fibroblasts upon stimulation by TNF-α all play different roles in exacerbating the disease, by promoting local inflammation and recruiting immune cells (34). Our findings support that TNF-α and -β play an important role in the interaction between B-cells and fibroblasts in our experimental models, which does not necessarily imply that anti-TNF-α treatment would be efficient in patients with SSc. Indeed, the role of anti TNF alpha treatment remains controversial in SSc. Although there have been no randomized controlled studies on anti-TNF drugs in patients with SSc or SSc-ILD, observational studies from the early 2000s suggested that anti-TNF drugs such as infliximab and etanercept might improve inflammatory arthritis and disability in patients with SSc (35, 36). A recent survey shows that 1/3 of patients treated in a center in Japan received anti-TNF (37). Moreover, other cytokines may also potentiate the effect of TNF-α, as shown *in vitro* for IFN-γ (38). In this work as well as in the literature, the effect of B-cells on fibroblasts was partially dampened by the use of a transwell system separating both cell types (10). Thus, while cytokines, and particularly TNF-α, seem to play an important role in B-cells/fibroblasts interactions, direct cell-cell contacts are also likely involved.

Single cell RNA sequencing showed an interesting difference in keratinocyte expression profile with or without 3D coculture with SSc A B-cells. We found DEGs related to keratinization and differentiation, two domains impaired in the skin of SSc patients (39). Of note, the use of tofacitinib to treat SSc reduces the expression of IFN-related genes in both cutaneous fibroblasts and keratinocytes, with the intensity of the effect depending on the differentiation state of keratinocytes (40).

Due to the small number of patients sharing the same clinical characteristics (skin extension, autoantibodies profile…) in a single experimental condition, we were not able to perform subgroup analysis for SSc type or autoantibody specificity. No difference was observed in our results based on SSc disease duration (data not shown).

In conclusion, the infiltration of B-cells observed in the human skin in SSc was reproduced *in vitro* in this 3D coculture model. SSc NA B-cells infiltrated more than HC NA B-cells and activation of B-cells increased the infiltration. We showed an impact both on B-cells and on fibroblasts of the coculture. Infiltrated B-cells from patients with SSc showed an activated profile and an overexpression of immunoglobulin genes compared to circulating B-cells before infiltration. We showed that activated B-cells modified the transcriptomic profile of both healthy and SSc fibroblasts, toward a pro-inflammatory (TNF and IL-17 signaling) and interferon profile, with a key role of the TNF pathway. As a perspective, it would be interesting to extend this study with a pathologic 3D skin model using fibroblasts from SSc patients and assess the role of TNF and COX inhibitors in this model. Altogether, our results reinforce and further decipher the role of B-cells in SSc and provide potential targets for future therapeutic approach.

## Data Availability

The datasets presented in this study can be found in online repositories. The names of the repository/repositories and accession number(s) can be found below: NCBI SRA, accession PRJNA1110840.
