# Supplementary material for: Characteristics and impact of infiltration of B-cells from systemic sclerosis patients in a 3D healthy skin model
Source: Front Immunol. 2024 Aug 9;15:1373464. doi: 10.3389/fimmu.2024.1373464 (PMC11341436; doi:10.3389/fimmu.2024.1373464)
Supplement: Supplementary file 1 [file DataSheet_1.pdf]

Supplementary Material

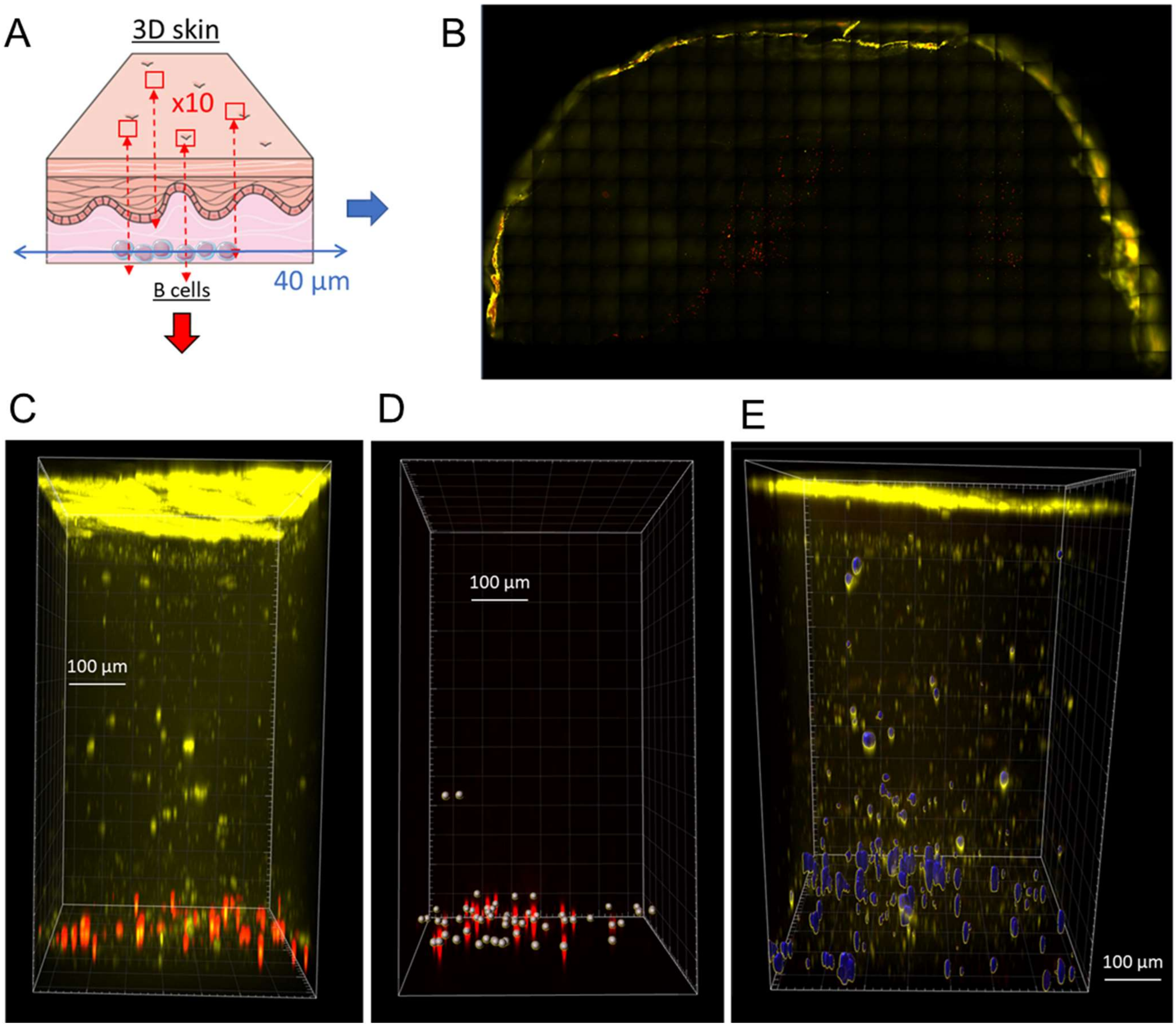

Supplementary Figure 1 – Approach for acquisition and analysis of confocal microscopy images on 3D cocultures. (A) Schematic representation detailing the method of image acquisition for each coculture sample. (B) Overview image (10X magnification, shown in blue) capturing the entire surface of the skin at a depth of 40 µm from the base of the dermis. (C) Acquisitions of 10 slices images covering the complete thickness of the skin (20X magnification, shown in red). Within these images, (D) the locations of B-cells are highlighted as white spheres, and (E) the volume occupied by the B-cell signal is denoted in blue. Skin areas are marked in yellow, B-cells are indicated in red. Analysis conducted using the 3D Imaris® software. Panel A was partly generated using Servier Medical Art.

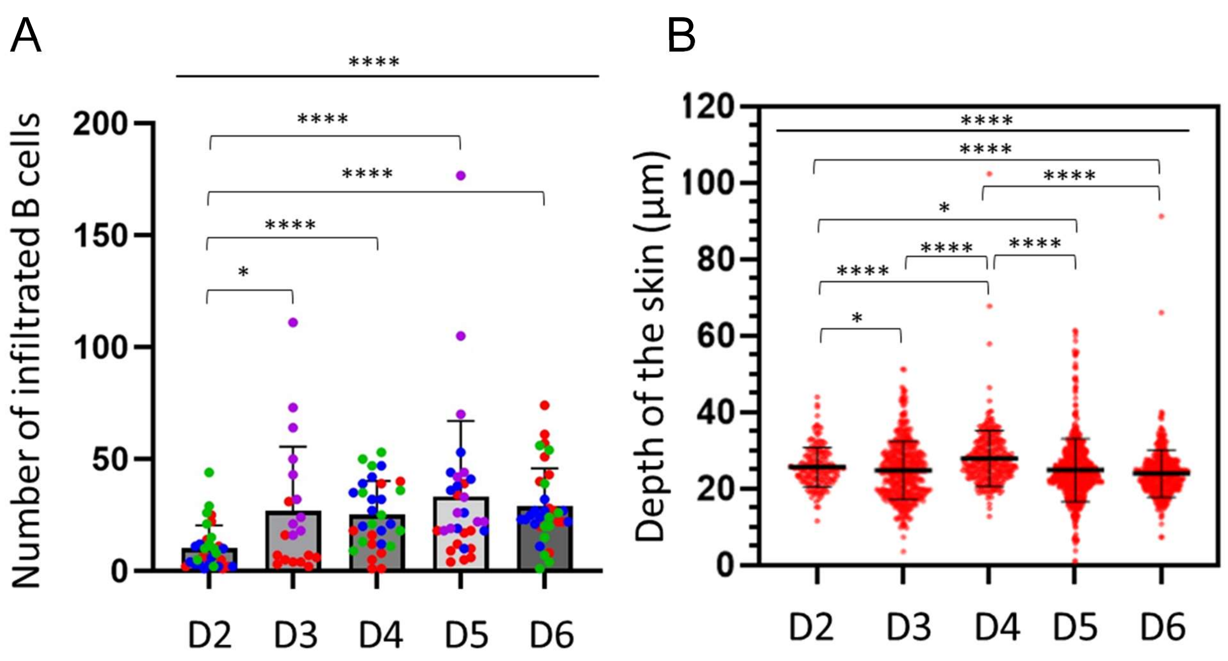

Supplementary Figure 2 – Kinetics of healthy activated B-cells infiltration in a 3D healthy skin model. (A) B-cell infiltration between 2 and 6 days of coculture quantified by the number of identified infiltrated B-cells per microscopy image. Each color represents a given B-cell donor. (B) Mean depth of infiltration between 2 and 6 days of coculture. Measures realized using z-stack experiment of the full thickness of the skin by confocal microscopy. Kruskal-Wallis non-parametric test (bar) and Mann-Whitney unpaired t-test (bracket). Mean +/- SD. *p<=0.05; **p<=0.01; ***p<=0.001; ****p<=0.0001. N = 2-3 cocultures per time point and 10 to 12 images per coculture.

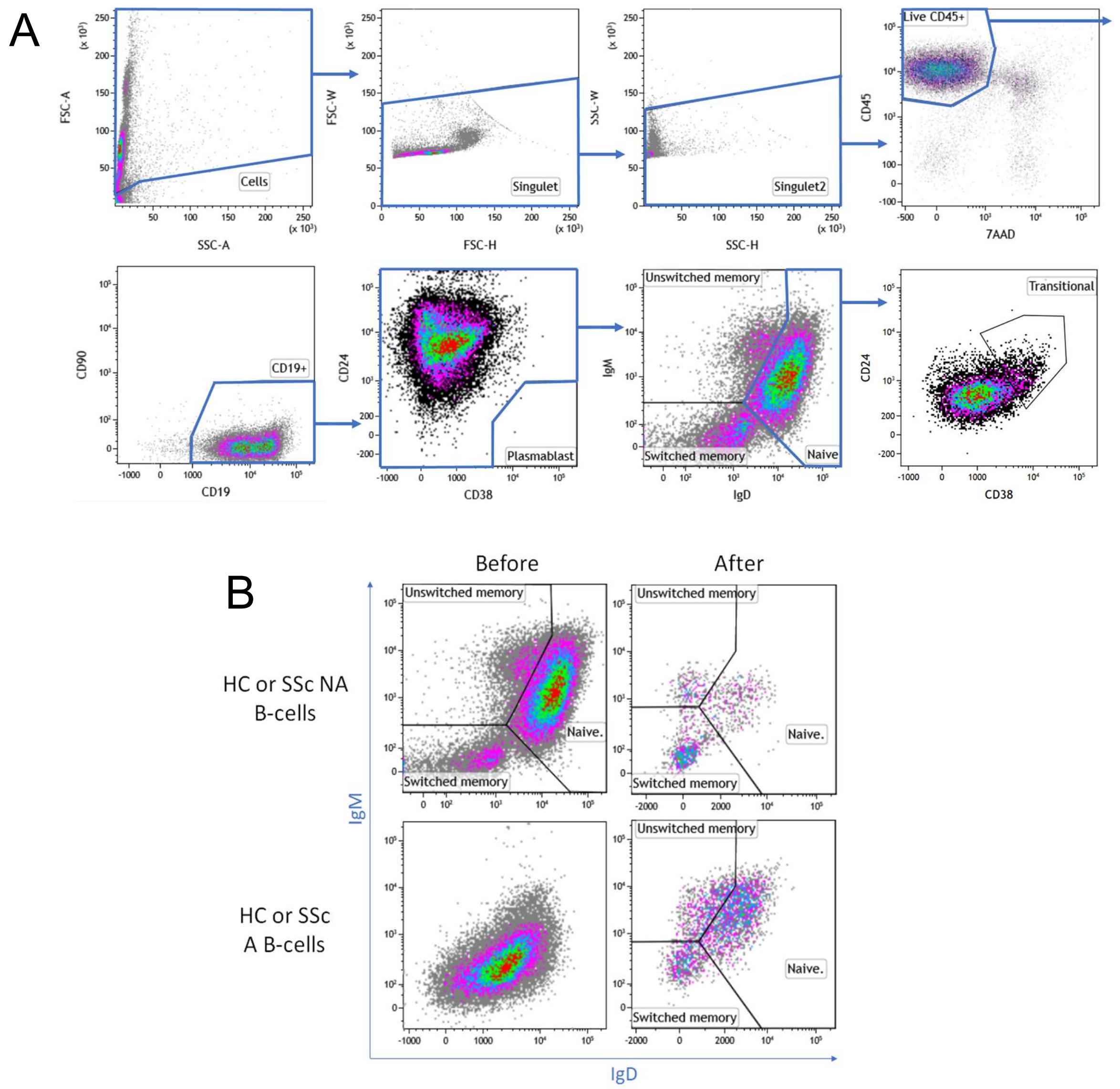

Supplementary Figure 3 – Cytometry analysis of B-cells before and after 3D cocultures. (A) Gating strategy for identification of B-cell sub-populations. (B) Representative IgM vs IgD profiles of non-activated and activated B-cells before and after coculture with 3D skin.

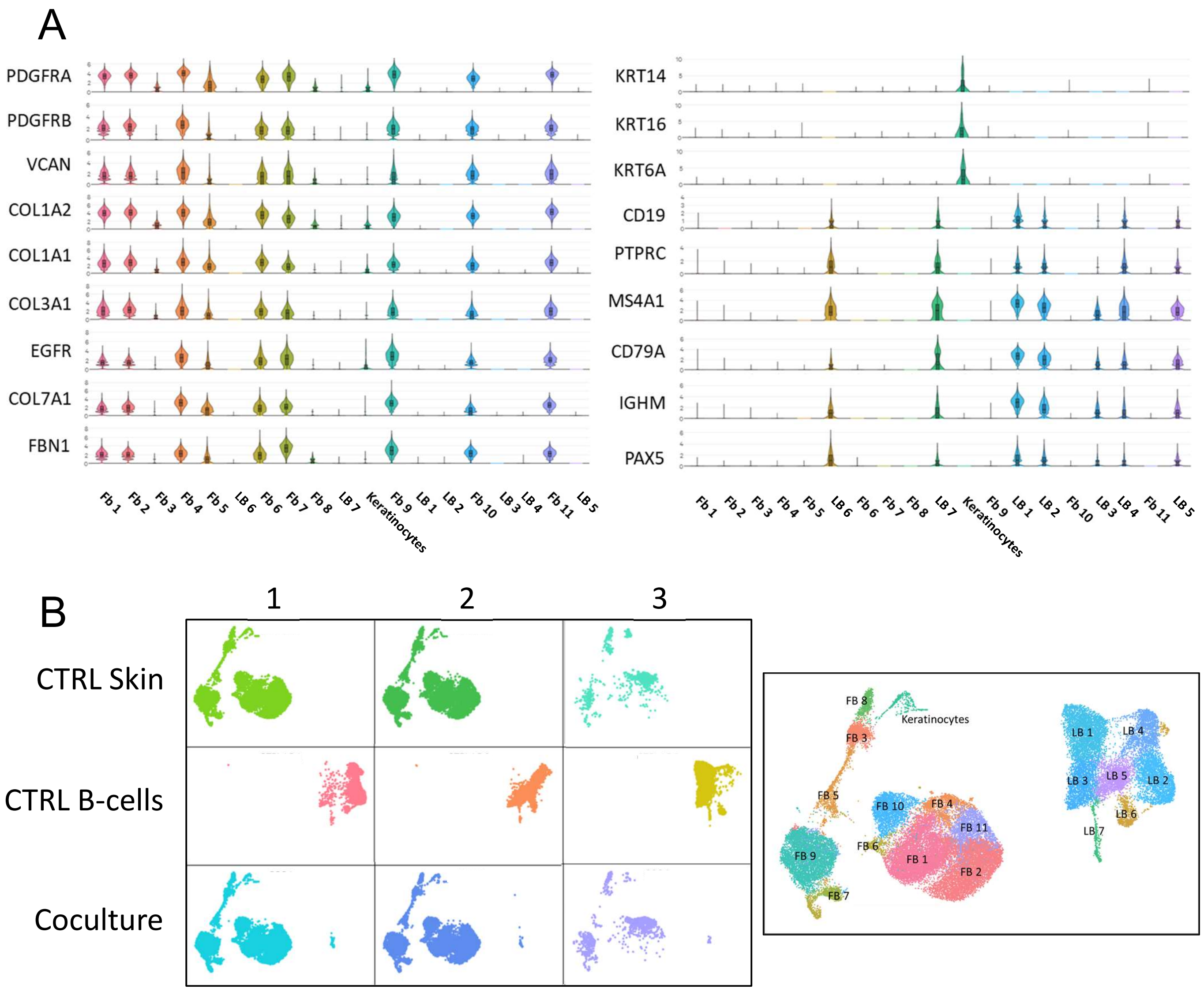

Supplementary Figure 4 – Identification of single-cell RNAseq clusters for 3D cocultures. (A) Markers used for identification of cell clusters. (B) UMAP visualization of single-cell RNAseq split by sample.

A

KEGG Pathway hsa04670 Leukocyte transendothelial migration

actin gamma 1(ACTG1)71
actin beta(ACTB)60
Rac family small GTPase 1(RAC1)5879

B

KEGG Pathway hsa05415 Diabetic cardiomyopathy

NADH dehydrogenase subunit 4(ND4)4538
ATP synthase F1 subunit delta(ATP5F1D)513
ATP synthase F0 subunit 8(ATP8)4509
cytochrome c oxidase subunit (COX1)4512
cytochrome c oxidase subunit (COX3)4514
cytochrome c oxidase subunit (COX2)4513
ubiquinol-cytochrome c reduct(UQCRH)7388
transforming growth factor be(TGFB1)7040

C

KEGG Pathway hsa05416 Viral myocarditis

major histocompatibility comp(HLA-DPB1)3115
major histocompatibility comp(HLA-DPA1)3113
major histocompatibility comp(HLA-DQB1)3119
major histocompatibility comp(HLA-DRB5)3127
major histocompatibility comp(HLA-DRA)3122
major histocompatibility comp(HLA-DMA)3108
major histocompatibility comp(HLA-A)3105
CD74 molecule(CD74)972
CD81 molecule(CD81)975
coronin 1A(CORO1A)11151

D

GO:0006955 immune response

GENE NAME
C-X-C motif chemokine receptor 4(CXCR4)
CD74 molecule(CD74)
CD79b molecule(CD79B)
cysteine rich protein 1(CRIP1)
immunoglobulin heavy constant alpha 1(IGHA1)
immunoglobulin heavy variable 1-2(IGHV1-2)
immunoglobulin heavy variable 3-23(IGHV3-23)
immunoglobulin kappa variable 1-5(IGKV1-5)
immunoglobulin kappa variable 3-15(IGKV3-15)
immunoglobulin kappa variable 3-20(IGKV3-20)
immunoglobulin lambda variable 2-14(IGLV2-14)
interferon induced transmembrane protein 2(IFITM2)
major histocompatibility complex, class I, A(HLA-A)
major histocompatibility complex, class I, C(HLA-C)
major histocompatibility complex, class II, DM alpha(HLA-DMA)
major histocompatibility complex, class II, DP alpha 1(HLA-DPA1)
major histocompatibility complex, class II, DP beta 1(HLA-DPB1)
major histocompatibility complex, class II, DQ beta 1(HLA-DQB1)
major histocompatibility complex, class II, DR alpha(HLA-DRA)
major histocompatibility complex, class II, DR beta 5(HLA-DRB5)

E

GO:0050853 B cell receptor signaling pathway

GENE NAME
BLK proto-oncogene, Src family tyrosine kinase(BLK)
CD79a molecule(CD79A)
CD79b molecule(CD79B)
immunoglobulin heavy constant alpha 1(IGHA1)
immunoglobulin heavy constant mu(IGHM)
immunoglobulin heavy variable 1-2(IGHV1-2)
immunoglobulin heavy variable 3-23(IGHV3-23)

F

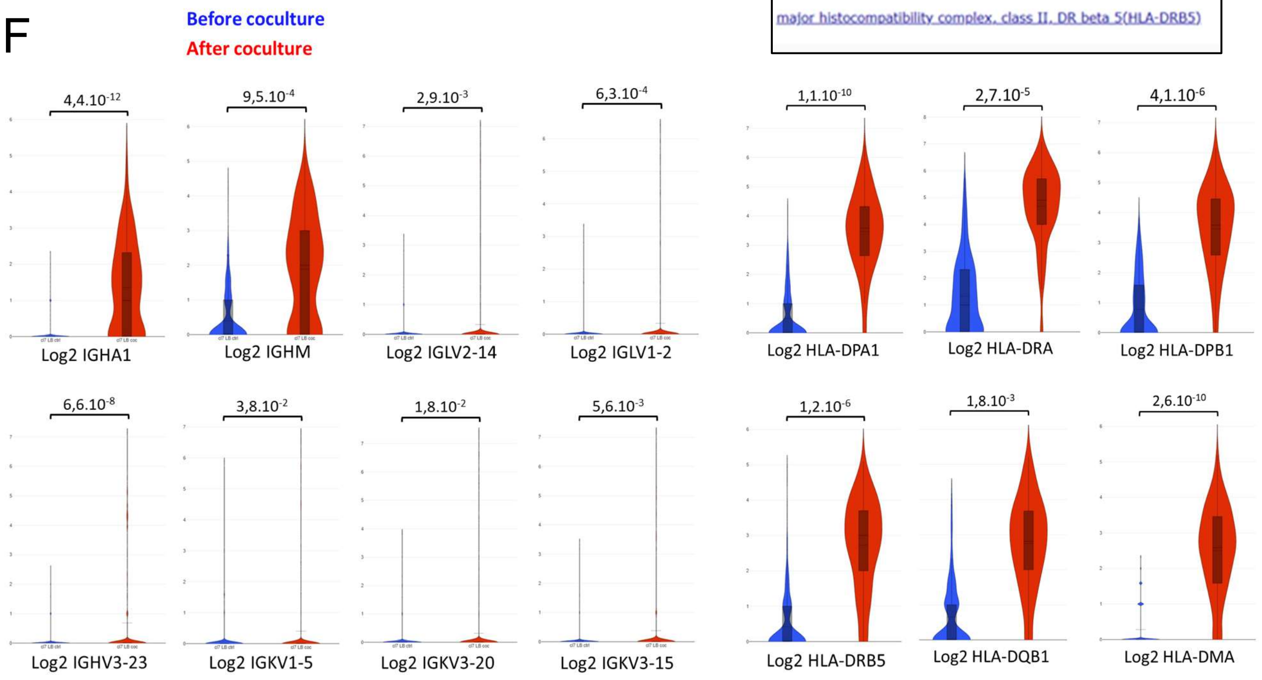

Supplementary Figure 5 – Gene lists from the enrichment analysis of upregulated genes in infiltrated activated SSc B-cells from 3D cocultures. Detail of genes associated with GO terms and KEGG pathways identified in Figure 3d : (A) KEGG Pathway hsa04670 "Leukocyte transendothelial migration". (B) KEGG Pathway hsa05415 "Diabetic cardiomyopathy". (C) KEGG Pathway hsa05416 "Viral myocarditis". (D) GOTERM GO:0006955 “Immune response” and (E) GO:0050853 " B cell receptor signaling pathway".
(F) Expression levels of Ig and HLA genes upregulated by cells from the LB7 cluster after coculture vs before coculture.

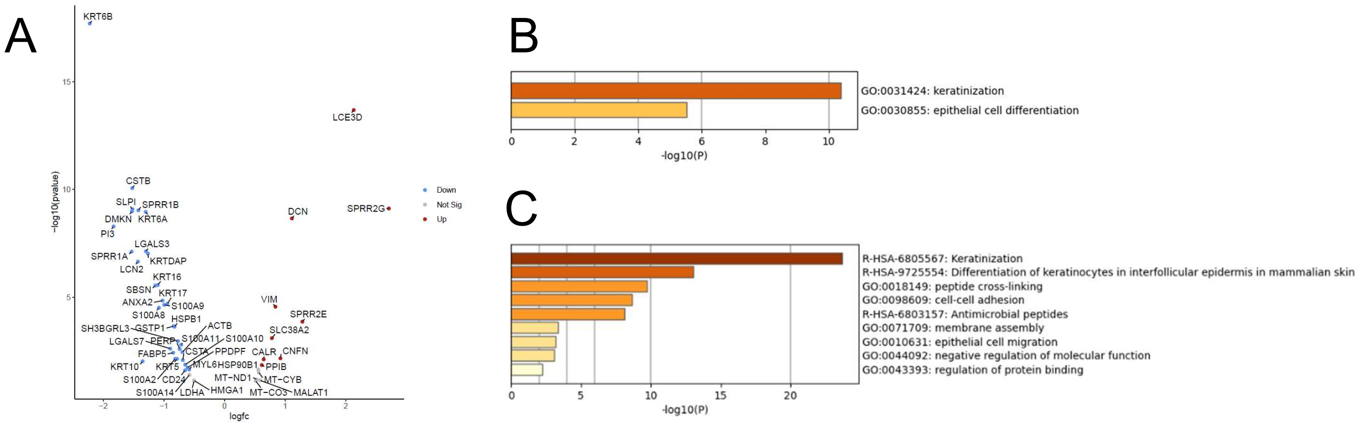

Supplementary Figure 6 – Enrichment analysis of genes differentially expressed in keratinocytes from 3D cocultures with SSc A B-cells vs ctrl 3D skin. (A) DEGs between keratinocytes from cocultures vs keratinocytes from control skin models analyzed by single cell RNAseq (thresholds : |FC] > 1,5 ; $p < 0.05$). An enrichment analysis was performed for 10 upregulated genes (B) and 33 downregulated genes (C) using Metascape.

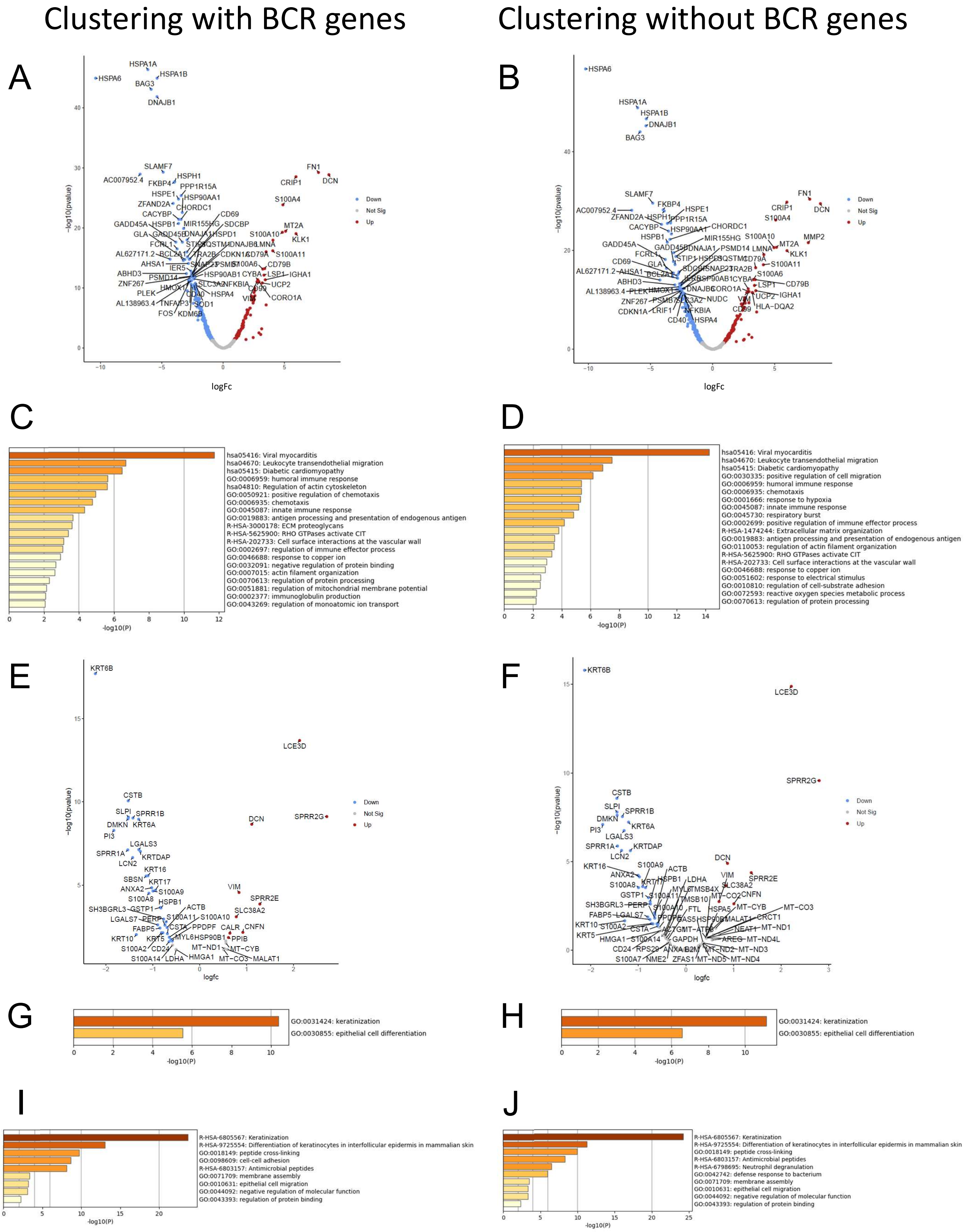

Clustering with BCR genes
Clustering without BCR genes
A
B
-log10(pvalue)
logFc
Down
Not Sig
Up
C
hsa05416: Viral myocarditis
hsa04670: Leukocyte transendothelial migration
hsa05415: Diabetic cardiomyopathy
GO:0006959: humoral immune response
hsa04810: Regulation of actin cytoskeleton
GO:0050921: positive regulation of chemotaxis
GO:0006935: chemotaxis
GO:0045087: innate immune response
GO:0019883: antigen processing and presentation of endogenous antigen
R-HSA-3000178: ECM proteoglycans
R-HSA-5625900: RHO GTPases activate CIT
R-HSA-202733: Cell surface interactions at the vascular wall
GO:0002697: regulation of immune effector process
GO:0046688: response to copper ion
GO:0032091: negative regulation of protein binding
GO:0007015: actin filament organization
GO:0070613: regulation of protein processing
GO:0051881: regulation of mitochondrial membrane potential
GO:0002377: immunoglobulin production
GO:0043269: regulation of monoatomic ion transport
-log10(P)
D
hsa05416: Viral myocarditis
hsa04670: Leukocyte transendothelial migration
hsa05415: Diabetic cardiomyopathy
GO:0030335: positive regulation of cell migration
GO:0006959: humoral immune response
GO:0006935: chemotaxis
GO:0001666: response to hypoxia
GO:0045087: innate immune response
GO:0045730: respiratory burst
GO:0002699: positive regulation of immune effector process
R-HSA-1474244: Extracellular matrix organization
GO:0019883: antigen processing and presentation of endogenous antigen
GO:0110053: regulation of actin filament organization
R-HSA-5625900: RHO GTPases activate CIT
R-HSA-202733: Cell surface interactions at the vascular wall
GO:0046688: response to copper ion
GO:0051602: response to electrical stimulus
GO:0010810: regulation of cell-substrate adhesion
GO:0072593: reactive oxygen species metabolic process
GO:0070613: regulation of protein processing
-log10(P)
E
F
-log10(pvalue)
logfc
Down
Not Sig
Up
G
H
GO:0031424: keratinization
GO:0030855: epithelial cell differentiation
-log10(P)
I
R-HSA-6805567: Keratinization
R-HSA-9725554: Differentiation of keratinocytes in interfollicular epidermis in mammalian skin
GO:0018149: peptide cross-linking
GO:0098609: cell-cell adhesion
R-HSA-6803157: Antimicrobial peptides
GO:0071709: membrane assembly
GO:0010631: epithelial cell migration
GO:0044092: negative regulation of molecular function
GO:0043393: regulation of protein binding
-log10(P)
J
R-HSA-6805567: Keratinization
R-HSA-9725554: Differentiation of keratinocytes in interfollicular epidermis in mammalian skin
GO:0018149: peptide cross-linking
R-HSA-6803157: Antimicrobial peptides
R-HSA-6798695: Neutrophil degranulation
GO:0042742: defense response to bacterium
GO:0071709: membrane assembly
GO:0010631: epithelial cell migration
GO:0044092: negative regulation of molecular function
GO:0043393: regulation of protein binding
-log10(P)

Supplementary Figure 7 – Single cell data results with or without including BCR genes during clustering. Differentially expressed genes between B-cells after coculture vs before coculture in cluster LB7 (A, B) and enrichment in Goterms, Interactome and KEGG among upregulated genes in LB7 after coculture (C, D). Differentially expressed genes between keratinocytes after coculture vs before coculture (E, F) and enrichment in Goterms, Interactome and KEGG among upregulated (G, H) and downreulated (I, J) genes in keratinocytes after coculture. Results of the analysis with BCR genes are presented on the left, and without BCR genes on the right.

A

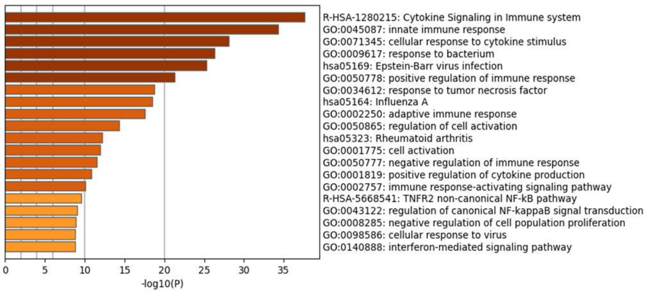

B

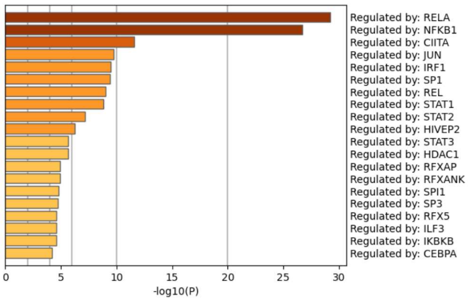

C

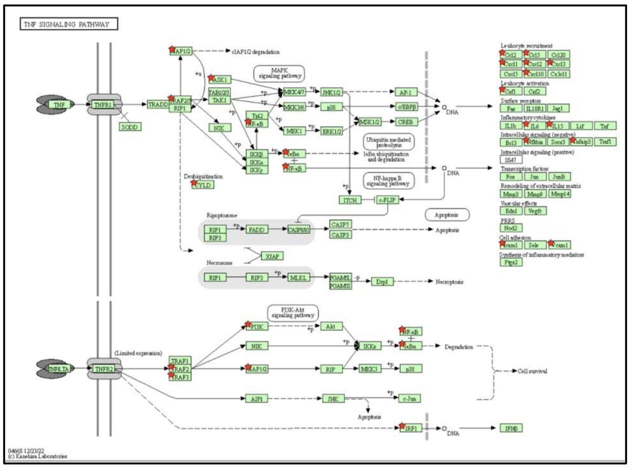

D

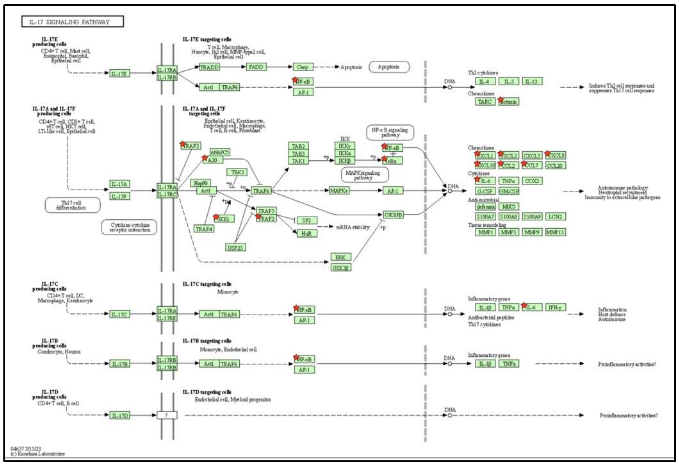

E

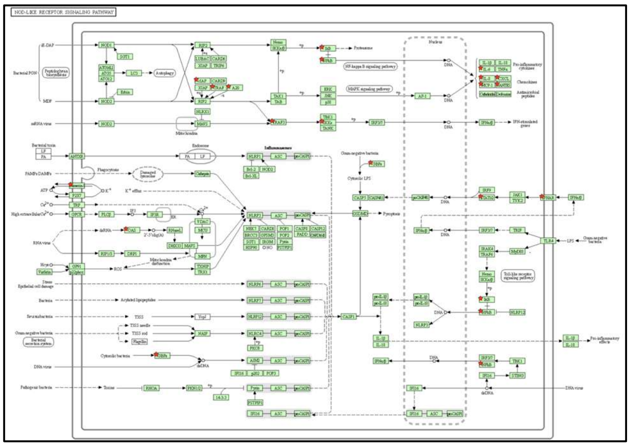

F

GENE NAME
C-C motif chemokine ligand 11(CCL11)
C-C motif chemokine ligand 13(CCL13)
C-C motif chemokine ligand 2(CCL2)
C-C motif chemokine ligand 5(CCL5)
C-C motif chemokine ligand 7(CCL7)
C-X-C motif chemokine ligand 1(CXCL1)
C-X-C motif chemokine ligand 10(CXCL10)
C-X-C motif chemokine ligand 11(CXCL11)
C-X-C motif chemokine ligand 3(CXCL3)
C-X-C motif chemokine ligand 8(CXCL8)
C-X-C motif chemokine ligand 9(CXCL9)
RELB proto-oncogene, NF-kB subunit(RELB)
TNF alpha induced protein 3(TNFAIP3)
TNF superfamily member 4(TNFSF4)
TNFAIP3 interacting protein 1(TNIP1)
TNFAIP3 interacting protein 3(TNIP3)
adhesion G protein-coupled receptor E5(ADGRE5)
apolipoprotein L3(APOL3)
bradykinin receptor B1(BDKRB1)
colony stimulating factor 1(CSF1)
complement C3(C3)
galectin 9(LGALS9)
guanylate binding protein 5(GBP5)
histone deacetylase 9(HDAC9)
interleukin 15(IL15)
interleukin 34(IL34)
interleukin 6(IL6)
nuclear factor kappa B subunit 1(NFKB1)
nuclear factor kappa B subunit 2(NFKB2)
phosphatidylinositol-4,5-bisphosphate 3-kinase catalytic subunit delta(PIK3CD)
phospholipase A2 group IVC(PLA2G4C)
sphingosine kinase 1(SPHK1)
vascular cell adhesion molecule 1(VCAM1)
zinc finger CCCH-type containing 12A(ZC3H12A)

G

intercellular adhesion molecu(ICAM1)3383
interleukin 32(IL32)9235
C-C motif chemokine ligand 11(CCL11)6356
galectin 9(LGALS9)3965
superoxide dismutase 2(SOD2)6648
vascular cell adhesion molecu(VCAM1)7412
signal transducer and activat(STAT1)6772
proteasome activator subunit (PSME2)5721
interleukin 6(IL6)3569
lymphocyte cytosolic protein (LCP1)3936
nuclear factor kappa B subuni(NFKB2)4791
colony stimulating factor 1(CSF1)1435
C-C motif chemokine ligand 2(CCL2)6347
tumor protein p53(TP53)7157
C-C motif chemokine ligand 5(CCL5)6352
C-X-C motif chemokine ligand (CXCL1)2919
C-X-C motif chemokine ligand (CXCL8)3576
interleukin 18 binding protei(IL18BP)10068
TNF receptor associated facto(TRAF2)7186
proteasome 20S subunit beta 9(PSMB9)5698
NFKB inhibitor alpha(NFKBIA)4792
interleukin 7 receptor(IL7R)3575
nuclear factor kappa B subuni(NFKB1)4790
interleukin 27 receptor subun(IL27RA)9466
interleukin 34(IL34)146433
phosphatidylinositol-4,5-bisp(PIK3CD)5293
interleukin 15(IL15)3600
proteasome activator subunit (PSME1)5720
C-X-C motif chemokine ligand (CXCL10)3627
sequestosome 1(SQSTM1)8878

H

C-X-C motif chemokine ligand (CXCL1)2919
C-X-C motif chemokine ligand (CXCL11)6373
C-X-C motif chemokine ligand (CXCL8)3576
C-X-C motif chemokine ligand (CXCL3)2921
C-X-C motif chemokine ligand (CXCL9)4283
C-X-C motif chemokine ligand (CXCL10)3627
C-C motif chemokine ligand 2(CCL2)6347
C-C motif chemokine ligand 13(CCL13)6357
C-C motif chemokine ligand 5(CCL5)6352
C-C motif chemokine ligand 7(CCL7)6354
C-C motif chemokine ligand 11(CCL11)6356

I

2'-5'-oligoadenylate syntheta(OAS2)4939
2'-5'-oligoadenylate syntheta(OAS3)4940
signal transducer and activat(STAT1)6772
interferon regulatory factor (IRF1)3659
guanylate binding protein 5(GBP5)115362
PML nuclear body scaffold(PML)5371
guanylate binding protein 1(GBP1)2633
interferon alpha inducible pr(IFI27)3429
interferon induced protein wi(IFIT5)24138
interferon induced protein wi(IFIT3)3437
bone marrow stromal cell anti(BST2)684
interferon alpha and beta rec(IFNAR2)3455
interferon stimulated exonucl(ISG20)3669
SAM and HD domain containing (SAMHD1)25939
interferon alpha inducible pr(IFI6)2537

Supplementary Figure 8 - Enrichment analysis of genes expressed by SSc fibroblasts in 2D cocultures with activated SSc B-cells compared to SSc fibroblasts alone. Analysis of the 200 most upregulated genes in the comparison with David and Metascape tools : (A) Enrichment in GO terms, KEGG and molecular pathways. (B) TRRUST analysis. (C) KEGG « TNF signaling pathway » Red stars mark genes from the list that are involved in the different pathways. (D) KEGG « IL-17 signaling pathway ». (E) KEGG « NOD-like receptor signaling pathway ». Detailed gene lists are provided for (F) GOTERM « Inflammatory response », (G) REACTOME R-HSA-1280215 « Cytokine Signaling in Immune system », (H) GOTERM GO:0071345 « Cellular response to cytokine stimulus », and (I) GOTERM GO:0045087 « Innate immune response ».

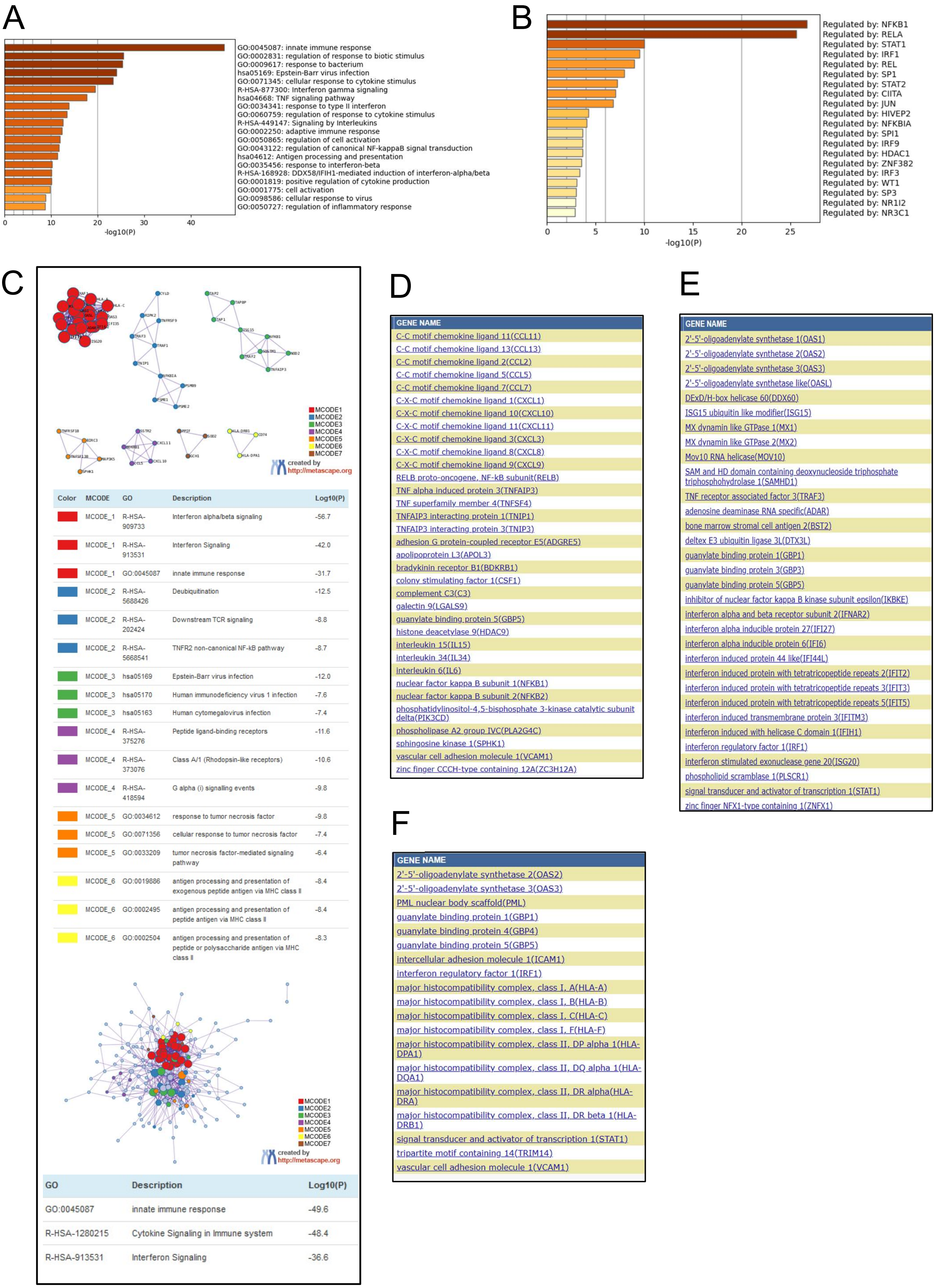
A
GO:0045087: innate immune response
GO:0002831: regulation of response to biotic stimulus
GO:0009617: response to bacterium
hsa05169: Epstein-Barr virus infection
GO:0071345: cellular response to cytokine stimulus
R-HSA-877300: Interferon gamma signaling
hsa04668: TNF signaling pathway
GO:0034341: response to type II interferon
GO:0060759: regulation of response to cytokine stimulus
R-HSA-449147: Signaling by Interleukins
GO:0002250: adaptive immune response
GO:0050865: regulation of cell activation
GO:0043122: regulation of canonical NF-kappaB signal transduction
hsa04612: Antigen processing and presentation
GO:0035456: response to interferon-beta
R-HSA-168928: DDX58/IFIH1-mediated induction of interferon-alpha/beta
GO:0001819: positive regulation of cytokine production
GO:0001775: cell activation
GO:0098586: cellular response to virus
GO:0050727: regulation of inflammatory response
0 10 20 30 40
-log10(P)
B
Regulated by: NFKB1
Regulated by: RELA
Regulated by: STAT1
Regulated by: IRF1
Regulated by: REL
Regulated by: SP1
Regulated by: STAT2
Regulated by: CIITA
Regulated by: JUN
Regulated by: HIVEP2
Regulated by: NFKBIA
Regulated by: SPI1
Regulated by: IRF9
Regulated by: HDAC1
Regulated by: ZNF382
Regulated by: IRF3
Regulated by: WT1
Regulated by: SP3
Regulated by: NR1I2
Regulated by: NR3C1
0 5 10 15 20 25
-log10(P)
C
MCODE1
MCODE2
MCODE3
MCODE4
MCODE5
MCODE6
MCODE7
created by http://metascape.org
Color | MCODE | GO | Description | Log10(P)
MCODE_1 | R-HSA-909733 | Interferon alpha/beta signaling | -56.7
MCODE_1 | R-HSA-913531 | Interferon Signaling | -42.0
MCODE_1 | GO:0045087 | innate immune response | -31.7
MCODE_2 | R-HSA-5688426 | Deubiquitination | -12.5
MCODE_2 | R-HSA-202424 | Downstream TCR signaling | -8.8
MCODE_2 | R-HSA-5668541 | TNFR2 non-canonical NF-kB pathway | -8.7
MCODE_3 | hsa05169 | Epstein-Barr virus infection | -12.0
MCODE_3 | hsa05170 | Human immunodeficiency virus 1 infection | -7.6
MCODE_3 | hsa05163 | Human cytomegalovirus infection | -7.4
MCODE_4 | R-HSA-375276 | Peptide ligand-binding receptors | -11.6
MCODE_4 | R-HSA-373076 | Class A/1 (Rhodopsin-like receptors) | -10.6
MCODE_4 | R-HSA-418594 | G alpha (i) signalling events | -9.8
MCODE_5 | GO:0034612 | response to tumor necrosis factor | -9.8
MCODE_5 | GO:0071356 | cellular response to tumor necrosis factor | -7.4
MCODE_5 | GO:0033209 | tumor necrosis factor-mediated signaling pathway | -6.4
MCODE_6 | GO:0019886 | antigen processing and presentation of exogenous peptide antigen via MHC class II | -8.4
MCODE_6 | GO:0002495 | antigen processing and presentation of peptide antigen via MHC class II | -8.4
MCODE_6 | GO:0002504 | antigen processing and presentation of peptide or polysaccharide antigen via MHC class II | -8.3
MCODE1
MCODE2
MCODE3
MCODE4
MCODE5
MCODE6
MCODE7
created by http://metascape.org
GO | Description | Log10(P)
GO:0045087 | innate immune response | -49.6
R-HSA-1280215 | Cytokine Signaling in Immune system | -48.4
R-HSA-913531 | Interferon Signaling | -36.6
D
GENE NAME
C-C motif chemokine ligand 11(CCL11)
C-C motif chemokine ligand 13(CCL13)
C-C motif chemokine ligand 2(CCL2)
C-C motif chemokine ligand 5(CCL5)
C-C motif chemokine ligand 7(CCL7)
C-X-C motif chemokine ligand 1(CXCL1)
C-X-C motif chemokine ligand 10(CXCL10)
C-X-C motif chemokine ligand 11(CXCL11)
C-X-C motif chemokine ligand 3(CXCL3)
C-X-C motif chemokine ligand 8(CXCL8)
C-X-C motif chemokine ligand 9(CXCL9)
RELB proto-oncogene, NF-kB subunit(RELB)
TNF alpha induced protein 3(TNFAIP3)
TNF superfamily member 4(TNFSF4)
TNFAIP3 interacting protein 1(TNIP1)
TNFAIP3 interacting protein 3(TNIP3)
adhesion G protein-coupled receptor E5(ADGRE5)
apolipoprotein L3(APOL3)
bradykinin receptor B1(BDKRB1)
colony stimulating factor 1(CSF1)
complement C3(C3)
galectin 9(LGALS9)
guanylate binding protein 5(GBP5)
histone deacetylase 9(HDAC9)
interleukin 15(IL15)
interleukin 34(IL34)
interleukin 6(IL6)
nuclear factor kappa B subunit 1(NFKB1)
nuclear factor kappa B subunit 2(NFKB2)
phosphatidylinositol-4,5-bisphosphate 3-kinase catalytic subunit delta(PIK3CD)
phospholipase A2 group IVC(PLA2G4C)
sphingosine kinase 1(SPHK1)
vascular cell adhesion molecule 1(VCAM1)
zinc finger CCCH-type containing 12A(ZC3H12A)
E
GENE NAME
2'-5'-oligoadenylate synthetase 1(OAS1)
2'-5'-oligoadenylate synthetase 2(OAS2)
2'-5'-oligoadenylate synthetase 3(OAS3)
2'-5'-oligoadenylate synthetase like(OASL)
DExD/H-box helicase 60(DDX60)
ISG15 ubiquitin like modifier(ISG15)
MX dynamin like GTPase 1(MX1)
MX dynamin like GTPase 2(MX2)
Mov10 RNA helicase(MOV10)
SAM and HD domain containing deoxynucleoside triphosphate triphosphohydrolase 1(SAMHD1)
TNF receptor associated factor 3(TRAF3)
adenosine deaminase RNA specific(ADAR)
bone marrow stromal cell antigen 2(BST2)
deltex E3 ubiquitin ligase 3L(DTX3L)
guanylate binding protein 1(GBP1)
guanylate binding protein 3(GBP3)
guanylate binding protein 5(GBP5)
inhibitor of nuclear factor kappa B kinase subunit epsilon(IKBKE)
interferon alpha and beta receptor subunit 2(IFNAR2)
interferon alpha inducible protein 27(IFI27)
interferon alpha inducible protein 6(IFI6)
interferon induced protein 44 like(IFI44L)
interferon induced protein with tetratricopeptide repeats 2(IFIT2)
interferon induced protein with tetratricopeptide repeats 3(IFIT3)
interferon induced protein with tetratricopeptide repeats 5(IFIT5)
interferon induced transmembrane protein 3(IFITM3)
interferon induced with helicase C domain 1(IFIH1)
interferon regulatory factor 1(IRF1)
interferon stimulated exonuclease gene 20(ISG20)
phospholipid scramblase 1(PLSCR1)
signal transducer and activator of transcription 1(STAT1)
zinc finger NFX1-type containing 1(ZNFX1)
F
GENE NAME
2'-5'-oligoadenylate synthetase 2(OAS2)
2'-5'-oligoadenylate synthetase 3(OAS3)
PML nuclear body scaffold(PML)
guanylate binding protein 1(GBP1)
guanylate binding protein 4(GBP4)
guanylate binding protein 5(GBP5)
intercellular adhesion molecule 1(ICAM1)
interferon regulatory factor 1(IRF1)
major histocompatibility complex, class I, A(HLA-A)
major histocompatibility complex, class I, B(HLA-B)
major histocompatibility complex, class I, C(HLA-C)
major histocompatibility complex, class I, F(HLA-F)
major histocompatibility complex, class II, DP alpha 1(HLA-DPA1)
major histocompatibility complex, class II, DQ alpha 1(HLA-DQA1)
major histocompatibility complex, class II, DR alpha(HLA-DRA)
major histocompatibility complex, class II, DR beta 1(HLA-DRB1)
signal transducer and activator of transcription 1(STAT1)
tripartite motif containing 14(TRIM14)
vascular cell adhesion molecule 1(VCAM1)

Supplementary Figure 9 - Enrichment analysis of genes expressed by SSc fibroblasts in 2D cocultures with activated SSc B-cells vs SSc fibroblasts in 2D cocultures with non-activated SSc B-cells. Analysis of the 200 most upregulated genes in the comparison with David and Metascape tools : (A) Enrichment in GO terms, KEGG and molecular pathways. (B) TRRUST analysis. (C) Protein-protein interactions network. Detailed gene lists are provided for (D) REACTOME R-HSA-913531 « Interferon Signaling » (E) GO:0051607 « Defense response to virus », and (F) REACTOME R-HSA-877300 « Interferon gamma signaling ».

A. Cytometry

	BEFORE						AFTER					
	Unswitched B-cells	Plasma-blasts	Switched B-cells	Naïve B-cells	Transitional B-cells	CD19+ B-cells	Unswitched B-cells	Plasma-blasts	Switched B-cells	Naïve B-cells	Transitional B-cells	CD19+ B-cells
LB HC NA							6	0	72	134	1	212
							12	0	60	342	0	416
							1	0	10	37	0	48
	2,926	2	5,530	19,569	2,592	28,044	2	0	94	184	0	281
	3,966	7	4,225	25,888	4,310	34,123	29	0	161	531	6	724
	707	0	1,567	5,694	469	7,977	13	0	221	460	0	695
	6,886	0	2,853	10,865	1,232	20,620	4	0	37	123	0	164
	4,021	2	2,080	14,102	2,188	20,268	33	0	137	524	0	691
LB HC A							36	0	161	750	26	950
							57	0	50	450	4	558
							500	0	578	2,518	40	3,606
							653	0	225	2,237	26	3,119
							292	0	256	526	1	1,082
							48	0	313	1,248	6	1,611
							15	0	73	176	10	265
							99	0	152	532	12	787
LB SSc NA	882	13	1,192	9,293	1,039	11,557	134	0	455	1,528	39	2,121
	948	3	1,207	4,656	154	7,103	49	0	239	635	0	925
	3,035	8	3,198	2,850	236	9,970	244	0	707	401	4	1,362
							210	0	84	37	0	335
	1,360	3	780	4,582	120	6,908	118	0	341	688	0	1,152
	4,866	20	2,588	25,564	2,286	33,422	313	0	1,146	821	8	2,291
	1,369	1	2,018	1,324	13	4,722	110	0	374	177	0	664
	519	0	1,014	3,508	183	5,057	38	0	243	312	3	595
LB SSc A							472	0	313	1,998	21	2,784
							297	0	150	1,946	103	2,392
							337	0	343	602	3	1,287
							625	0	639	4,164	106	5,435
							793	0	599	1,805	30	3,205
							379	2	922	1,655	61	2,966
							2 152	4	1,546	4,752	97	8,465

B. Single-cell RNAseq

	Skin			B-cells			Coculture			
	1	2	3	1	2	3	1	2	3	Total
Fb 1	1,704	1,731	55				1,851	1,786	73	7,200
Fb 2	1,488	1,549	28				1,699	1,624	30	6,418
Fb 3	338	339	62				272	266	32	1,309
Fb 4	22	23	558				33	17	616	1,269
Fb 5	193	235	16				242	205	23	914
Fb 6	102	194	39				162	135	35	667
Fb 7	129	145	7				157	137	22	597
Fb 8	79	85	73				104	91	76	508
Fb 9	1,181	1,244	131				1,108	994	214	4,872
Fb 10	455	520	60				575	566	84	2,260
Fb 11	440	411	15				455	390	43	1,754
Keratinocytes	82	63	55				91	91	21	403
LB 1				39	20	3,436				3,495
LB 2				2,492	25	13				2,530
LB 3				84	421	1,404				1,909
LB 4				974	677	115				1,766
LB 5				42	1,574	88				1,704
LB 6				162	263	264				689
LB 7				58	129	86	127	50	23	473
Total	6,213	6,539	1,099	3,851	3,109	5,406	6,876	6,352	1,292	40,737

Supplementary Table 1 - Cell counts for cytometry (A) and single-cell RNAseq (B) experiments. Grey cells represent in (A) unavailable data due to technical issues, and in (B) clusters absent from the samples.
